# Performance evaluation of CuBTC composites for room temperature oxygen storage†

**DOI:** 10.1039/d0ra07068h

**Published:** 2020-11-10

**Authors:** Leena Melag, M. Munir Sadiq, Kristina Konstas, Farnaz Zadehahmadi, Kiyonori Suzuki, Matthew R. Hill

**Affiliations:** Department of Chemical Engineering, Monash University Clayton VIC 3168 Australia Matthew.Hill@csiro.au; Department of Materials Science and Engineering, Monash University Clayton VIC 3168 Australia; CSIRO Private Bag 33, Clayton South MDC VIC 3169 Australia

## Abstract

Oxygen is commonly separated from air using cryogenic liquefaction. The inherent energy penalties of phase change inspire the search for energy-efficient separation processes. Here, an alternative approach is presented, where we determine whether it is possible to utilise simpler, stable materials in the right process to achieve overall energy efficiency. Adsorption and release by Metal–Organic Frameworks (MOFs) are an attractive alternative due to their high adsorption and storage capacity at ambient conditions. Cu-BTC/MgFe_2_O_4_ composites were prepared, and magnetic induction swing adsorption (MISA) used to release adsorbed oxygen quickly and efficiently. The 3 wt% MgFe_2_O_4_ composites exhibited an oxygen uptake capacity of 0.34 mmol g^−1^ at 298 K and when exposed to a magnetic field of 31 mT, attained a temperature rise of 86 °C and released 100% of adsorbed oxygen. This water vapor stable pelletized system, can be filled and emptied within 10 minutes requiring around 5.6 MJ kg^−1^ of energy.

## Abstract 2

Oxygen is commonly separated from air using cryogenic liquefaction.

## Graphical abstract

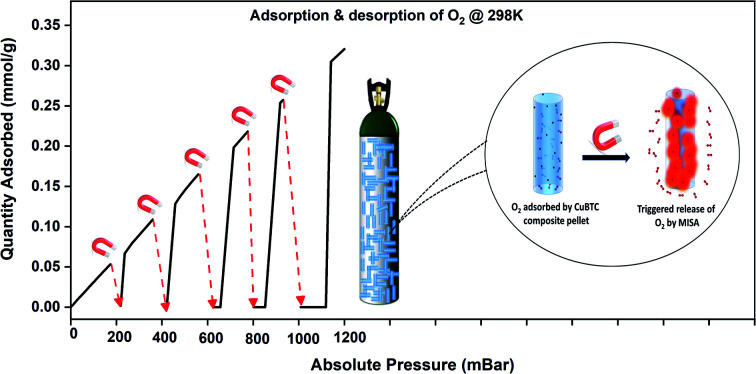

## 1. Introduction

The demand for high purity oxygen is on the rise owing to its increased consumption by the healthcare, steel, food, water, chemical, and pharmaceutical industries and this, in turn, has influenced research into more energy-efficient ways to capture, separate and store oxygen.^1,2^ The traditional process of cryogenic liquefication of air^3^ produces ultra-high purity oxygen and still dominates the industrial methods of oxygen separation, but the complex plant setups and the energy requirements associated with the entire process limits its use for large scale productions only. The membrane-based separations and adsorption-based processes using zeolites and carbon molecular sieves are simpler, reversible, low cost and easily scalable for small scale separations of oxygen, but their structural rigidity, pore heterogeneity, and low oxygen permeabilities impose limitations on the purity of the oxygen produced.^4^ Once produced, compressed or liquefied oxygen is bottled up in storage tanks for subsequent use in varied applications.^2,5–7^ Taking into account the safety hazards associated with the handling and storing of a highly reactive gas like oxygen, equal emphasis is required on finding alternate means to counter the current high-pressure (*ca.* 140 bar) storage of oxygen; a safe, lightweight alternative that would store oxygen in high volumes but at much lower pressures.^8–10^

Metal–Organic Frameworks (MOFs) are porous nanomaterials that have been explored for gas separations,^11–14^ catalysis,^15^ sensing,^11,16–23^ and drug delivery applications.^24,25^ They are constructed using metal nodes and organic linkers resulting in unique modular structures that are flexible, porous and offer the distinctive chemical tunability needed for gas storage.^26–28^ Their ability to host functional nanoparticles within their structures imparts added diverse functionalities to their existing versatile properties.^29^ Similar to most adsorption processes, separations in MOFs are based on the guest–host interactions where the adsorbents can either physically adsorb gas molecules on its surface or can bind to them chemically.^30^ For selective separations using MOFs, the selectivity between the different gas molecules relies on preferential size-selective sieving or favourable quadrupole interactions or strong chemical affinities with either of the adsorbates.^31^ Most oxygen separation processes are nitrogen selective and are based on the adsorption or isolation of nitrogen from the air to separate oxygen. However, using MOFs, selective oxygen separation has been studied through a process that relies on oxygen molecules directly binding to the metal cations in the framework leading to higher selectivity over other gases, mainly nitrogen.^32–35^

Accordingly, to investigate the role of MOFs in oxygen separation and storage, DeCoste *et al.* conducted simulation studies on 10 000 hypothetical MOFs and recorded NU-125 as the MOF with the highest oxygen adsorption capacity of 17.4 mol kg^−1^ at 140 bar pressure. Computational studies by Moghadam *et al.* on existing 2932 MOFs reported UMCM-152 as the MOF with a deliverable volumetric oxygen capacity of 249 cm^3^(STP) cm^−3^ and gravimetric oxygen storage of 19.6 mmol kg^−1^ obtained at 140 bar storage and 5 bar release pressures at 298 K. Similarly, various other MOFs like IRMOFs, UiO-66, Cr_3_(BTC)_2_,^33,35,36^ Cr-BTT,^37^ and M_2_(dobdc) (M = Cr, Mn, Fe, Co), especially Co_2_(dobdc)^38–40^ and Fe_2_(dobdc)^35,41,42^ have been investigated in detail as oxygen selective adsorbents for separation and storage. Apart from specially designed and developed MOFs, the existing range of MOFs need to be explored for a simpler, stable, and recyclable solution for room temperature oxygen storage. This paper investigates a widely used copper-based MOF, CuBTC, also known as HKUST-1 or MOF-199, previously identified by both DeCoste *et al.* and Moghadam *et al.* for oxygen storage applications. Owing to its affinity towards oxygen, CuBTC MOFs have been used as cathode catalysts in fuel cell technology that is impeded by a sluggish oxygen reduction reaction (ORR) and catalytic efficiency, cost, and stability. With its large surface area and a large number of sites for the catalytic reactions, CuBTC MOFs have been studied to replace platinum as the non-noble metal ORR catalyst or have been used as the sacrificial template for carbon-based electrocatalysts.^43–47^ CuBTC (copper(ii) benzene-1,3,5-tricarboxylate), is one of the widely explored, easily scalable, and most commercially used MOFs in various applications. Its ease of synthesis, high surface areas (1500–2000 m^2^ g^−1^), and excellent thermal and structural stability make it applicable for gas adsorption,^48–52^ separation,^49,53–56^ and sensors^17,57,58^ applications.

Temperature Swing Adsorption (TSA) and Pressure Swing Adsorption (PSA) are the most commonly used regeneration processes for MOFs. However, their strong host–guest interactions with the adsorbed molecules, particularly the strong adsorption at lower partial pressures and their thermally insulating nature, limit the uniform transfer of the applied heat throughout the MOF, making the regeneration process very challenging and energy-intensive.^11,59–63^ To address this, our group has demonstrated the potentials of incorporating stimuli-responsive materials in MOFs to achieve energy-efficient release of the trapped gases.^11,39,59,60,64–68^ In this paper, we discuss how rapid and remote heat generation can be achieved through the fabrication of Magnetic Framework Composites (MFCs) and how their interactions with a magnetic field can be used efficiently for release of the adsorbed molecules. Magnetic Induction Swing Adsorption (MISA) is a magnetically induced heating process aimed at regeneration of MOFs.^69–71^ In our previous paper, we have demonstrated the efficiency of the MISA process by desorption of 4.8 mmol g^−1^ of adsorbed oxygen from Co-MOF-74/Fe_3_O_4_ systems at 204 K and 1 bar pressure.^39^ To build upon these results obtained at cryogenic temperatures of 204 K, we intend to explore the possibility of simpler, stabler, cyclable MOFs for oxygen adsorption at room temperature. This paper looks into the relative stability and capacity of CuBTC MOF for oxygen storage, and the feasibility of oxygen release using MISA at room temperatures and 1 bar pressure.

The exposure of CuBTC MFC pellets formed using 3 wt% of MgFe_2_O_4_ nanoparticles, to a magnetic field of 33 mT, at a frequency of 269 kHz, triggered a 100% release of the 0.30 mmol g^−1^ oxygen molecules stored at 1 bar pressure. The ease of use and control of the MISA process was demonstrated by triggering an on-demand release of oxygen at 200, 400, 600, 800, and 1000 mbar pressures and achieving 100% desorption each time. Three continuous cycles of adsorption and magnetically triggered desorption cycles helped to establish the structural rigidity, thermal stability, and adsorption capacity of the CuBTC MFC. The effect of atmospheric exposure, and the effect of exposure to water vapour on the structural stability of the MFCs, was also investigated.

## 2. Experimental

### 2.1 Materials synthesis

All the reagents including 1,3,5-benzenetricarboxylic acid (H_3_BTC), copper acetate monohydrate Cu(OAc)_2_·H_2_O, sodium acetate trihydrate (CH_3_COONa·3H_2_O), magnesium chloride hexahydrate (MgCl_2_·6H_2_O), ferric chloride hexahydrate (FeCl_3_·6H_2_O), PEG and the solvents, *N*,*N*-dimethylformamide (DMF) and ethanol used for the synthesis were of analytical grade, obtained from commercial vendors and used as received.

#### Synthesis of CuBTC MOF

0.7 g of copper acetate monohydrate (3.5 mmol) dissolved in 14 mL of deionized water was mixed with 0.6 g of trimesic acid (H_3_BTC, 2.8 mmol) dissolved in 14 mL of ethanol. This mixture was stirred for 30 min, transferred into an autoclave, and heated to 85 °C for 24 h. On cooling, the blue CuBTC MOF was washed three times with ethanol and dried in a vacuum oven at 140 °C for 24 h^55,72^

#### Synthesis of MgFe_2_O_4_ nanoparticles

MgFe_2_O_4_ nanoparticles were synthesized using a solvothermal method by mixing of 3.6 g, 0.027 moles of sodium acetate trihydrate, 2.5 mmol MgCl_2_·6H_2_O and 5 mmol FeCl_3_·6H_2_O together and adding 2.00 g of polyethylene glycol (MW = 4000) as a surfactant. The mixture is stirred vigorously to form a homogeneous solution and then heated under reflux at 180 °C for 16 h. On cooling, the black magnetic nanoparticles are magnetically separated and washed alternately with distilled water and ethanol and dried in a vacuum oven at 80 °C for 8 h^73^

#### Fabrication of CuBTC pellets

The objective behind the shaping of MOFs is to pack maximum amounts of adsorbents in the storage tank compactly, to increase the amount of gas stored per unit volume. For practical applications, shaping MOFs is favourable. Still, the shaping technique should not adversely affect the stability and or adsorption capacity of the MOFs, and it should be feasible for large scale productions too. The most common shaping technique, pelletising, can be achieved by either applying a certain amount of pressure on the powdered MOFs to shape them into pellets or by mixing the MOF powder with specific binders (typically, polyvinyl alcohol (PVA)) and solvent to make a paste that can be further extruded into pellets.^55,72,74^ The Cu-BTC/MgFe_2_O_4_ MFCs were pelletised by extruding a paste made using measured quantities of CuBTC MOF, binder, and MgFe_2_O_4_ nanoparticles, through a 5 mL syringe. The extruded MFC noodles were cut into 8–10 mm pellets and allowed to dry in ambient air before drying them in a vacuum oven at 140 °C for 24 h. To select an MFC having an optimal balance between adsorption capacities, heating abilities, and structural stability, different MFCs with varying binder concentrations (1, 2, 3, 4 wt%) and varying magnetic content (1, 2, 3, 4 wt% of MgFe_2_O_4_ nanoparticles) were fabricated and investigated for their surface area and oxygen adsorption properties.

### 2.2 Characterisation of materials

The samples were characterised using X-ray Powder Diffraction (XRD), Scanning Electron Microscopy (SEM), Energy Dispersive Spectroscopy (EDS) and thermogravimetric analysis (TGA). Fourier-Transform Infrared (FTIR) spectra for all samples were collected using a Thermo Scientific NICOLET 6700 FT-IR. XRD measurements analysed the crystal structure of the samples on a D8 ADVANCE Eco X-ray powder diffractometer with a Co K_α_ radiation source of 1.79 Å with a scan rate of 0.05 s per step at 40 kV and 25 mA. JOEL 7001F Scanning Electron Microscope was used for the morphological size-shape study of all the samples. The surface area measurements were carried out using Micromeritics ASAP 2420 instruments, and the oxygen adsorption studies were carried out on a 3Flex surface and catalyst characterisation instrument. For the triggered release experiments, the 3Flex was paired with a radio frequency power supply induction machine (EASY HEAT 0224–Ambrell) operated at 269 kHz with an 8 turns heating coil of 2.5 cm diameter and 4 cm in length.

## 3. Results and discussion

**Fig. 1:**
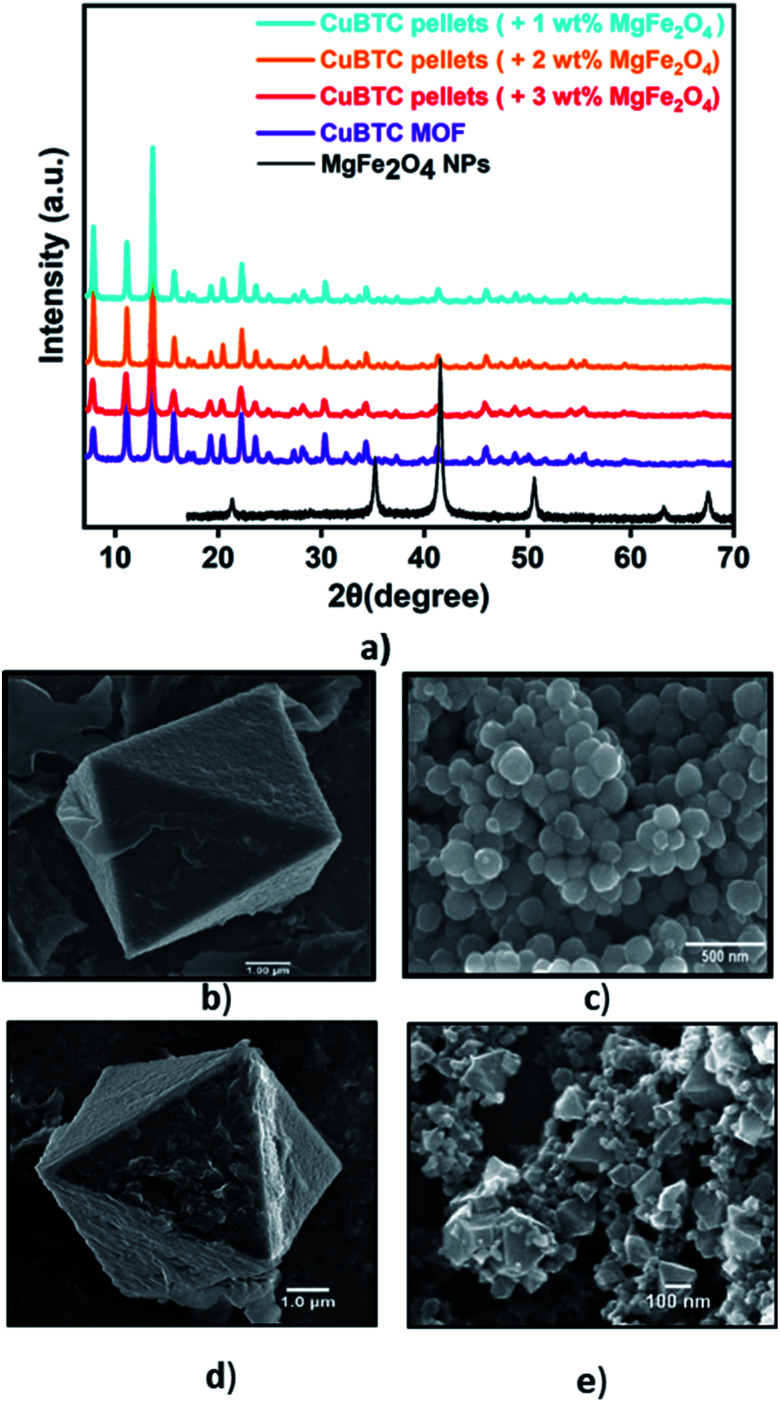
(a) The powder diffraction pattern of bare CuBTC MOF, MgFe_2_O_4_ nanoparticles, CuBTC–MgFe_2_O_4_ composite with 1 wt%, 2 wt%, 3 wt% magnetic content., and SEM images of (b) bare CuBTC (c) the MgFe_2_O_4_ nanoparticles (d) bare CuBTC (e) 3 wt% CuBTC–MgFe_2_O_4_ composite.

Fig. 1a presents the diffraction patterns of the synthesized powdered CuBTC MOF, the magnetic nanoparticles, and the MFCs fabricated with 1 wt%, 2 wt%, and 3 wt%, MgFe_2_O_4_ nanoparticles. The diffraction peaks from the synthesized CuBTC sample corresponds to the face centre cubic (FCC) CuBTC structure, which matches well with the peaks of the simulated CuBTC (Fig. S1a†). The clear, distinct sharp peaks confirm the excellent crystallinity of the sample. Due to their low concentrations, peaks corresponding to the magnetic nanoparticles are not visible in the composite XRD. The morphology of the bare CuBTC and composite was investigated with Scanning Electron Microscopy (SEM) analysis. SEM micrographs presented in Fig. 1b and d reveal octahedral shaped CuBTC particles with an average particle size of about 5 μm. Fig. 1c shows a uniform, spherical morphology for MgFe_2_O_4_ nanoparticles with a diameter of 150–170 nm. The nanoparticles were used to fabricate the MFCs by varying their concentrations during the pelletization process. Fig. 1e is a micrograph of the MFC with 3 wt%, MgFe_2_O_4_ nanoparticles, which reveals the magnetic nanoparticles firmly bound to the surfaces of the MOF particles and Fig. S2c–e† show its elemental distribution. The XRD analysis of the synthesized MgFe_2_O_4_ nanoparticles (Fig. S2a†) shows the diffraction peaks of planes (2 2 0), (3 1 1), (4 0 0), (5 1 1) and (4 4 0), for a cubic spinel MgFe_2_O_4_ phase, that matches the standard powder diffraction data (ICSD #00-036-0398) of the MgFe_2_O_4_ phase from literature^73^ with a calculated mean crystallite size of 20.2 nm.

Vibrating sample magnetometer (RIKEN DENSHI VSM) was used to study the response of MgFe_2_O_4_ nanoparticles to an applied magnetic field. When exposed to an applied magnetic field, the magnetization (*M*) of the MgFe_2_O_4_ nanoparticles increases with an increase in the magnetic field until it becomes saturated at 70 emu g^−1^ (Fig. S3a†). The Curie temperature (*T*_C_) of the MgFe_2_O_4_ nanoparticles, defined as the temperatures above which the ferrimagnetic nanoparticles become paramagnetic,^12,75–78^ was evaluated through thermo-magneto gravimetric analysis (TMGA) (Fig. S4†). This was estimated to be 566 °C. This agrees with *T*_C_ values reported in the literature for MgFe_2_O_4_.^59,66,69^ Specific adsorption rate, SAR, is a parameter used in estimating the magnitude of the heating effect generated by magnetic nanoparticles when exposed to an alternating current magnetic field.^11,59,66,69^ The SAR is a valuable parameter that can be used to estimate the rate of conversion of the magnetic field to thermal energy. A high SAR implies rapid heating, with SAR of the synthesized MgFe_2_O_4_ nanoparticles calculated to be 130 W g^−1^ under an applied field of 25 mT.

For gas adsorption measurements, the CuBTC samples were activated at 140 °C for 24 h, and the N_2_ adsorption isotherms obtained at 77 K show a type I adsorption that is typically observed in microporous solids. The calculated Brunauer–Emmett–Teller (BET) surface areas of the bare CuBTC MOF and MFCs are summarized in Fig. S5a and Table S1.† The BET surface area of the bare CuBTC MOF was 1495 m^2^ g^−1^, and upon addition of 1 wt% binder, a drop of 17% in surface area was noticed. This loss in surface area can be attributed to the process of pelletising where the binders, which are essential to enable cohesion and densification of the MOFs, cause partial blockage of some pores in the MOF resulting in lower surface areas and pore volumes.^79^ Based on such effects of different binder concentrations and varying magnetic contents on the surface area properties of the MFC, the Cu-BTC/MgFe_2_O_4_ composites with 3 wt% binder concentration and 3 wt% MgFe_2_O_4_ nanoparticles were selected and fabricated for all experiments.

To evaluate the potential of these Cu-BTC/MgFe_2_O_4_ MFCs for room temperature oxygen storage applications, it is crucial to establish their moisture stability in ambient atmospheric conditions.^80^ Water adsorption experiments were conducted to test the water stability of the MFCs using Micromeritics 3Flex gas sorption analyser for the range (*P*/*P*_o_ = 0.001–0.9) at 298 K. Prior to the vapour adsorption measurements, the CuBTC samples were activated at 140 °C for 12 h. The initial steep slope (0.001–0.2), the intermediate shallow plateau (0.2–0.7) and the last steep slope (0.7–0.9) of the water adsorption isotherm are all indicative of the strong interactions between the water molecules and the copper centres from the initial adsorption in the CuBTC cages to the final micropore filling of the side pockets. The water vapour adsorption capacity of 28.4 mmol g^−1^ of the CuBTC MOF matches well with the results reported in the literature.^50^ The water vapour adsorption capacity of the 3 wt% CuBTC–MgFe_2_O_4_ composite pellets was found to be 28.9 mmol g^−1^. Post water adsorption experiments, the structural stability of the MOF and MFC were analysed by powder X-ray diffraction (PXRD), and the matching XRD peak intensities of the activated samples and the hydrated samples confirm the structural stability (Fig. S6† and 2).

**Fig. 2:**
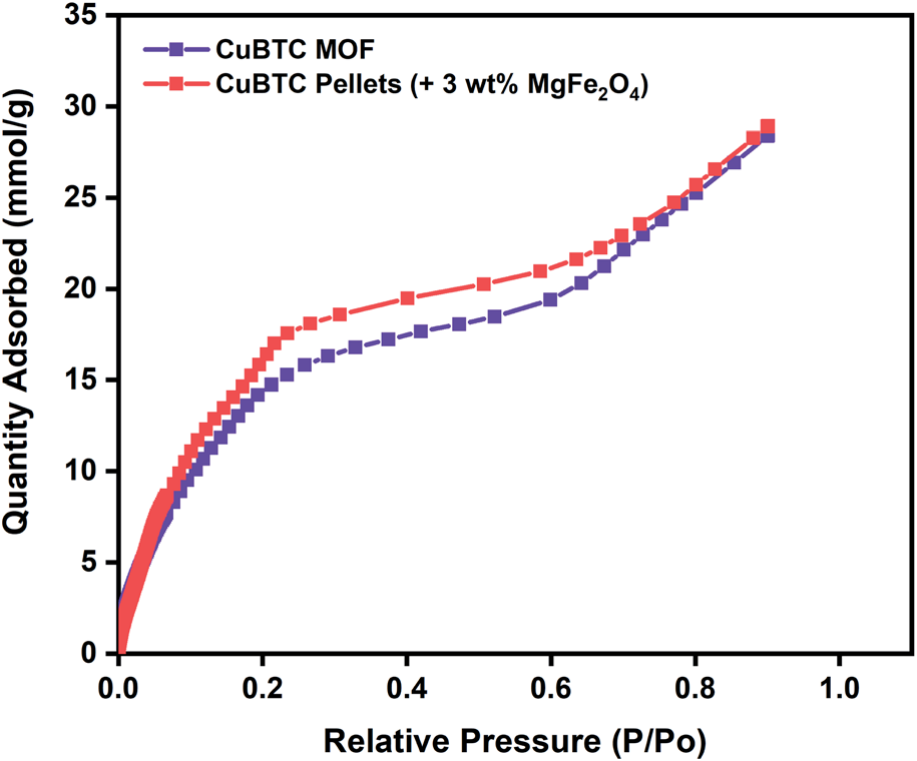
Water vapour adsorption isotherms of CuBTC MOF and 3 wt% CuBTC–MgFe_2_O_4_ composite pellets measured at 298 K.

### 3.1 Oxygen adsorption

**Fig. 3:**
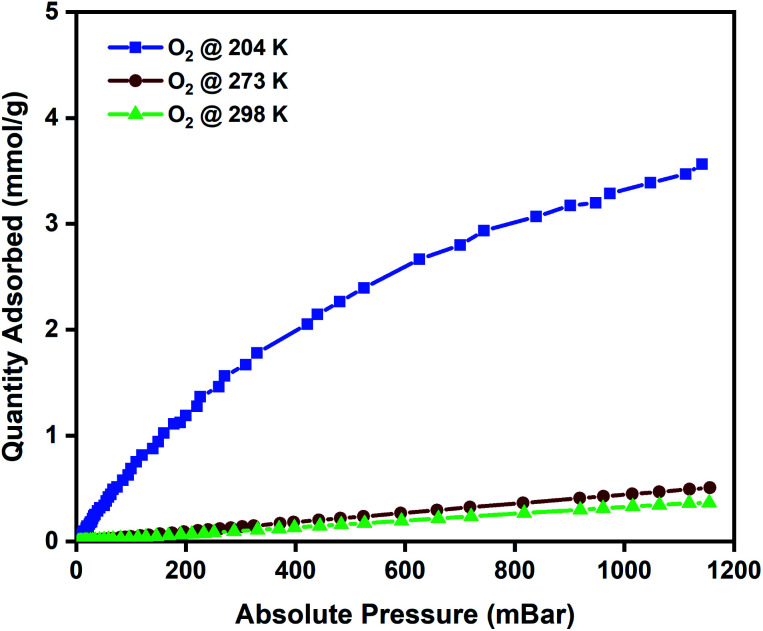
Oxygen adsorption isotherms of CuBTC MOF measured at 204, 273, and 298 K.

To evaluate the oxygen adsorption properties of the CuBTC pellets, single-component oxygen adsorption measurements were carried out at 204, 273, and 298 K (pressures loadings between 0 to 1 bar), on 80 mg of activated CuBTC pellets. The adsorption temperatures of 204, 273, and 298 K were stabilized using acetone and dry-ice bath, ice bath, and water bath, respectively. The results from the oxygen adsorption at these three temperatures reveal that at 204 K, the pellets displayed the highest adsorption capacity of 3.5 mmol g^−1^ of oxygen, followed by 0.45 mmol g^−1^ oxygen at 273 K and 0.34 mmol g^−1^ of oxygen was adsorbed at 298 K (Fig. 3). In CuBTC MOF,^13^ the dimeric copper-tetracarboxylic unit of Cu–Cu (2.628 Å) acts as a centre and is connected by four oxygen atoms from the benzenetricarboxylate (BTC) linkers and water molecules.^81–84^ The interconnected Cu(ii) paddlewheel unit and tridentate BTC linkers form a three-dimensional octahedral structure with square-shaped main channels of 9 × 9 Å and tetrahedral units of 5 Å openings that are connected to the main channels by triangular pockets of 3.5 Å. The isosteric heat of adsorption, *Q*_st_, reveals the extent of interaction between the adsorbed molecules and the adsorbate under constant loading conditions and here these interactions are primarily dependant on the reactions at the exposed cationic Cu^2+^ sites and adsorption at the windows sites of the octahedral CuBTC cage is calculated to be −15.3 kJ mol^−1^. This near-constant *Q*_st_ curve of oxygen was plotted using the adsorption data measured at 204, 273, and 298 K (Fig. S8b†) and shows that irrespective of the loading conditions the binding energies remain constant.

**Fig. 4:**
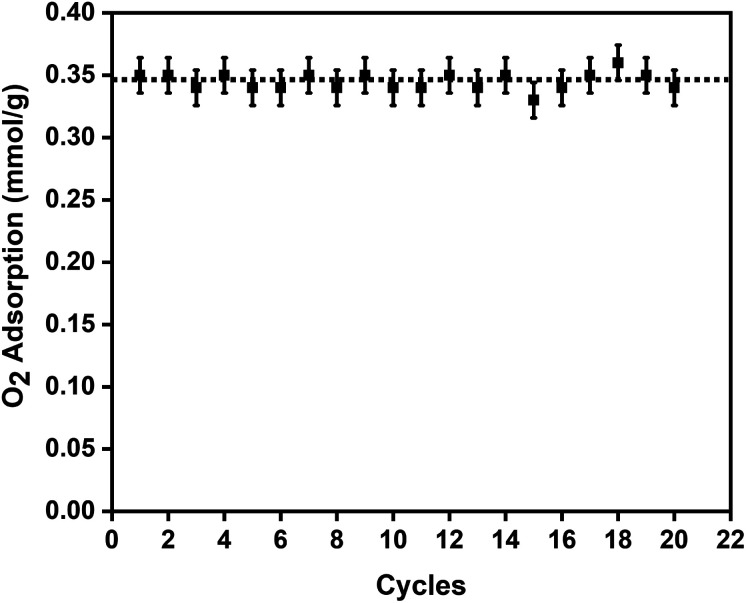
Twenty oxygen adsorption–desorption cycles on the 3 wt% CuBTC–MgFe_2_O_4_ composite pellets at 298 K with the error bars representing ±0.02 standard deviation per cycle.

To study the reversibility and reusability of the MFC pellets after their interactions with oxygen at room temperature, cyclic studies were carried out on the same sample. Between every oxygen adsorption cycle, they were regenerated at 140 °C for 12 h. As observed from Fig. 4, over twenty continuous cycles, the MFC pellets displayed a consistent oxygen adsorption capacity. They did not show any signs of material degradation even after the 20^th^ cycle, which was later confirmed by PXRD (Fig. S10†). Thermal stabilities and decomposition temperatures of the samples were also studied by thermal gravimetric analysis (TGA) using weighed samples that were heated from 25 °C to 800 °C at a heating rate of 10 °C min^−1^ (Fig. S7a†).

### 3.2 Desorption of oxygen using MISA

Magnetic induction swing adsorption is an innovative technology that harnesses the heating abilities of magnetic nanoparticles to trigger the release of stored gas molecules from the framework in energy-efficient ways. The adsorption potential of the MOFs and the heating abilities of the magnetic nanoparticles are combined to develop the MFCs. This is to enable efficient heat transfer in the MOF by overcoming their low thermal conductivities. It is achieved using ferrimagnetic nanoparticles capable of remote, targeted, and localized heat generation capabilities upon exposure to a magnetic field.^60,85,86^ The heat generation in the nanoparticles is a result of hysteresis observed in the plot of magnetisation *vs.* the applied field when the ferrimagnets are exposed to an alternating current magnetic field. An irreversible magnetisation–demagnetisation process is induced with the magnitude of the heat generated equivalent to the area within the hysteresis loop.^69,87,88^ Consequently, in MFCs, this rapid and localized heat generation leads to instability in the guest–host bond of the adsorbed gas and the framework that triggers the release of the gas molecules. The selection of the MOF, the ratio of magnetic nanoparticles, and the strength of the applied magnetic field are essential features of the MISA process. The heat generation capability of the MFCs was measured by recording their temperature rise profile while exposing them to different magnetic field strengths. While studying the 3 wt% Cu-BTC/MgFe_2_O_4_ MFCs, it was noted that on application of 25 mT magnetic field, from the initial temperatures of 25 °C, the pellets attained a maximum temperature rise of 78 °C, with 31 mT magnetic field the temperature reached was 86 °C and with 33 mT, the MFCs reached a temperature of 92 °C (Fig. S4d†).

**Fig. 5:**
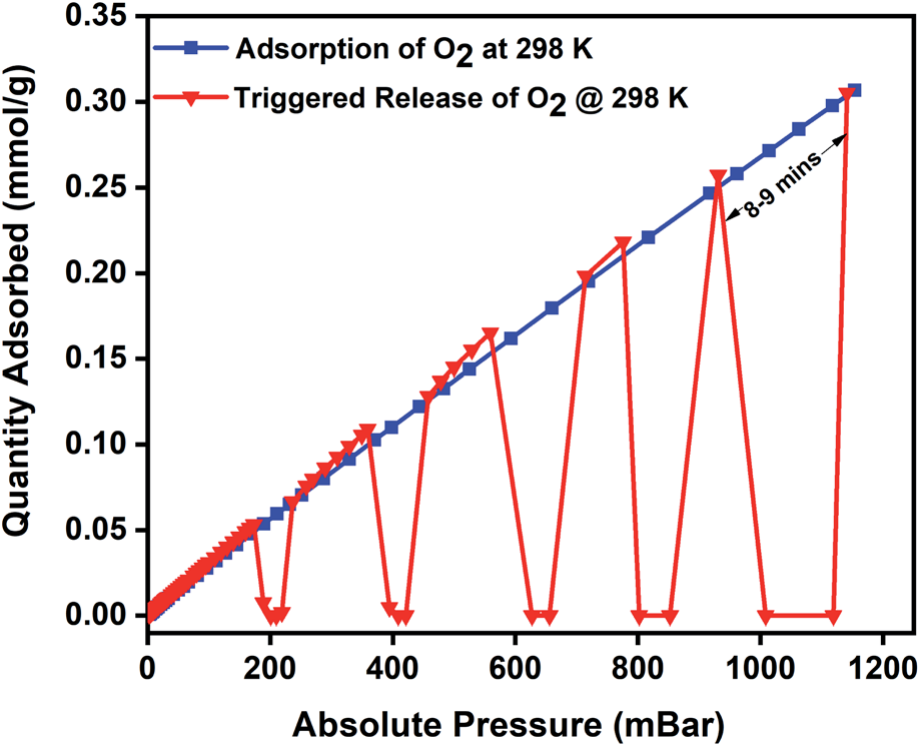
Oxygen adsorption isotherm of 3 wt% CuBTC–MgFe_2_O_4_ composite pellets at 298 K and the magnetically triggered desorption at 200, 400, 600, 800, and 1000 mbar.

The oxygen adsorption isotherms were collected using a Micro metrics 3Flex gas sorption analyser at pre-set equilibration times, allowing enough time for the system to equilibrate at each pressure point, and the targeted pressures for desorption were set at 200, 400, 600, 800, and 1000 mbar. The experiment was designed to alternate between adsorption and desorption phases continuously, with minimal activation and degassing taking place between each cycle. The desorption was induced with an EASY HEAT Ambrell induction machine (Fig. S9†) to trigger the remote, rapid, and localised heating enabling the complete release of the adsorbed oxygen at the desired pressure. Typically, the 3Flex experiment program is run to adsorb oxygen from 0.1 mbar to 1200 mbar. The experiment is monitored, and at 200 mbar pressure range, the remote magnetic heating is activated to trigger the release of the adsorbed oxygen molecules. The process is repeated at 400, 600, 800, and 1000 mbar. For the 3 wt% MFC, a magnetic field of 31 mT was applied, and at 188 mbar, a 100% release of the 0.06 mmol g^−1^ of adsorbed oxygen was released in 5 minutes. Once complete desorption was achieved, the applied magnetic field was switched off to stop the remote heating process, thereby allowing the MFC to resume the adsorption of oxygen. The MFC pellets showed a similar uptake performance relative to the bare MOF. The triggered release of the MFC was repeated at 400 mbar (0.12 mmol g^−1^), 600 mbar (0.17 mmol g^−1^), 800 mbar (0.22 mmol g^−1^), and 1000 mbar (0.26 mmol g^−1^) and achieved 100% release of oxygen molecules within 5 min time except for the 1000–1200 mbar range where it took 8–9 min for the adsorption–desorption–adsorption cycle to finish (Fig. 5). To investigate the post-MISA stability of the MFC, its regeneration, and adsorption capacities of the MFC pellets, they were activated at 140 °C for 6 h after each triggered release experiment, and after twenty MISA cycles (Fig. 4), the XRD results corroborate their structural stability(Fig. S10†).

**Fig. 6:**
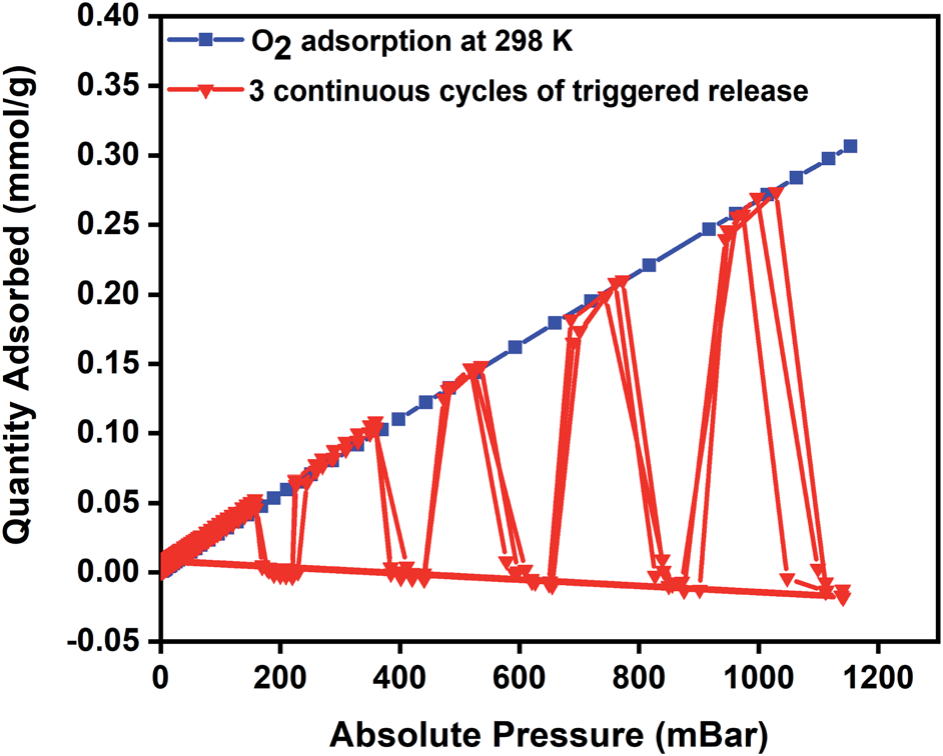
Oxygen adsorption isotherm of 3 wt% CuBTC–MgFe_2_O_4_ composite at 298 K and three continuous desorption cycles of oxygen at 200, 400, 600, 800, and 1000 mbar.

To evaluate regeneration capability and the cyclic performance of the composite pellets over multiple closed MISA cycles, upon achieving the maximum oxygen uptake of 0.34 mmol g^−1^ at 1140 mbar, the pellets were reactivated for 10 minutes by remote magnetic heating at 33 mT (92 °C) and simultaneously evacuating the sample from 1200–0 mbar. Fig. 6 presents the results of three continuous closed MISA cycles on the 3Flex with a sample pressure of 0.007 mbar achieved when the magnetic heating and vacuum activation steps were combined. Upon switching off the magnetic field, oxygen adsorption and MISA-based desorption second cycle continued precisely like the first cycle at 200, 400, 600, 800, and 1000 mbar and 31 mT (86 °C) applied magnetic field. The results from three continuous adsorptions and MISA regeneration of the MFCs revealed that the oxygen adsorption capacity of the composite pellets was not adversely affected by the heat from the nanoparticles and each desorption cycle achieved a consistent result of complete desorption of bound oxygen molecules.

**Fig. 7:**
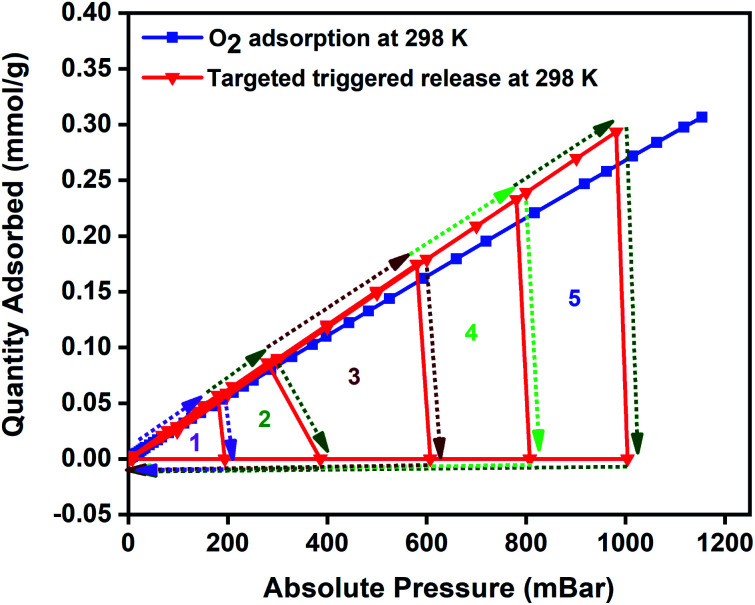
The performance of 3 wt% CuBTC–MgFe_2_O_4_ composite at 298 K in a closed MISA cyclic adsorption–desorption–regeneration process with the combined vacuum and magnetic heating triggered desorption at 200, 400, 600, 800 and 1000 mbar and the regeneration from 200–0 mbar (1), 400–0 mbar (2), 600–0 mbar (3), 800–0 mbar (4) and 1000–0 mbar (5).

To highlight the easy accessibility of oxygen at any targeted pressures and to signify the versatility of the MISA process in regenerating the MFC after every adsorption cycle, we ran a closed MISA adsorption–desorption–regeneration cyclic run across all pressures. This experiment was planned to alternate between adsorption, desorption, and regeneration between each cycle. The experiment was conducted with the usual oxygen adsorption by the MFCs, that was followed by desorption at 200 mbar using a magnetic field of 31 mT (86 °C), and the 200–0 mbar regeneration was achieved within 6 min by the magnetic heating from 33 mT (92 °C) magnetic field and evacuation from the system. Once 0 mbar pressure was reached, the MFC resumed its oxygen adsorption until the desorption and regeneration steps were activated again at 400, 600, 800, and 1000 mbar pressures. The regeneration times varied between 6–10 minutes with 200–0 mbar reached in 6 minutes, whereas it took 10 minutes for the 1200–0 mbar step. The 140 °C for 12 h heating plus vacuum reactivation step was replaced with 10 minutes, and 33 mT (92 °C) applied field at the end of each pressure point (downward-facing arrows at 1, 2, 3, 4 and 5 in Fig. 7). The success of this new adsorbent regeneration method can be validated by the consistent oxygen adsorption capacities shown by the composite in consecutive cycles.

### 3.3 Regeneration energy

For efficient and economic adsorption-based separations, the adsorbent selection is primarily governed by its selectivity, adsorption capabilities, regeneration ability, and stability. The regeneration energy is the energy utilized by the adsorbent to undo the adsorption process. It depends on the heat of adsorption, specific heat capacity, and working capacity of the adsorbents. It is directly proportional to the heat of adsorption because strong interaction between the adsorbent and adsorbate results in a higher heat of adsorption and consequently higher regeneration energy would be required to overcome this strong interaction^89^ leading to a high energy penalty.^11,89,90^ Furthermore, MOFs are generally known to be thermal insulators and require high temperatures to trigger the release of adsorbed species. The remote, localised and targeted heating nature of the MISA process makes it most suitable for oxygen capture and storage applications while minimising the energy penalty required to operate the process.^11,59,60,91^

**Fig. 8:**
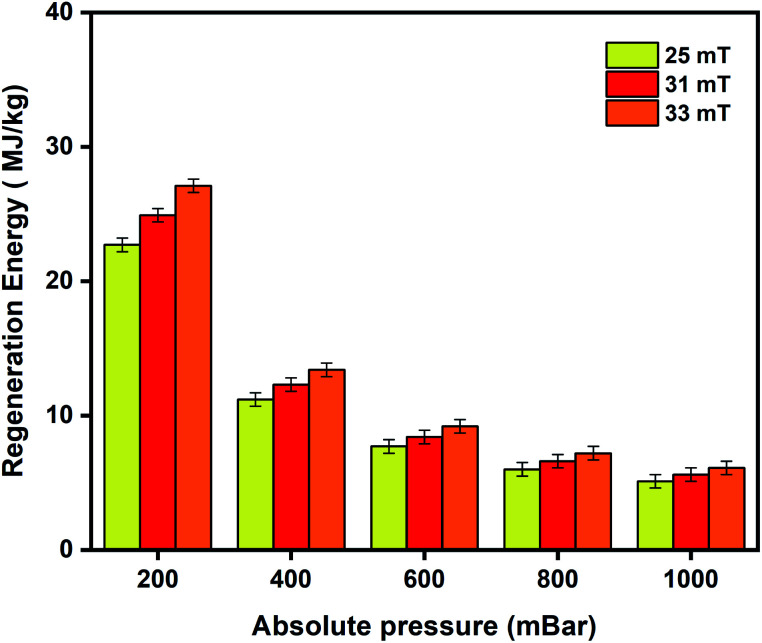
Regeneration energy as a function of varying magnetic fields of 25 mT, 31 mT, and 33 mT used to trigger desorption of oxygen from the 3 wt% CuBTC–MgFe_2_O_4_ MFCs.

Fig. 8 depicts the regeneration energy requirements with varying magnetic fields of 25 mT, 31 mT, and 33 mT. Stronger interactions between the adsorbed molecules and the framework at low pressures require higher regeneration energies for desorption at 200 mbar. Energy requirements by the MISA process were evaluated for two different masses of the MFC pellets. When 0.3 g and 0.6 g of the 3 wt% MFC pellets were exposed to a magnetic field of 31 mT; the MFC pellets experienced a temperature rise of 86 °C and the energy utilized for the regeneration of 0.26 mmol g^−1^ of oxygen adsorbed at 1000 mbar was calculated to be 5.1 MJ kg_O_2__^−1^ for 0.3 g weight sample and 5.6 MJ kg_O_2__^−1^ for 0.6 g sample (Fig. S11b†).

Additionally, the energy used to drive the magnetic induction heating in the MISA process was determined by monitoring the electrical power consumption of the EASY HEAT Ambrell induction machine. A power meter connected to the machine estimated the power consumption when the MFC is in and out of the magnetic field at 25 mT, 31 mT, and 33 mT. For the 0.6 g of the MFC pellets, the energy consumed to regenerate the pellets at 1000 mbar was calculated from the power (W) consumed by the induction unit, the time (s) it took to release the adsorbed molecules and the mass (g) of the released molecules (Table S3†). Despite the relatively high regeneration energy requirement of the MFC pellets, the energy input to the induction system was calculated to be 0.15 kW h kg_O_2__^−1^ (Table S2†) highlighting a lower energy requirement when compared to conventional cryogenic oxygen producing systems where energy requirement of 0.3–0.35 kW h kg_O_2__^−1^ has been reported.^92^

## 4. Conclusions

MOFs are known to be thermally insulating, and this can be overcome by utilizing the rapid, localized, and easily controllable magnetic induction heating effect. MISA is a technology that can be harnessed to achieve an efficient process for on-demand oxygen delivery. Particularly, miniaturized oxygen concentrators can be engineered with MFCs for room temperature oxygen storage and on-demand supply of oxygen. The results demonstrate the versatility and potential of the MISA process in exploiting a readily available MOF incorporated with magnetic nanoparticles for safe storage and on-demand release of oxygen at ambient conditions. Here, the powdered CuBTC MOF was transformed into CuBTC–MgFe_2_O_4_ MFCs by mixing them with different amounts of MgFe_2_O_4_ nanoparticles and pelletising them with the help of a binder. To understand the purity and properties of the synthesized CuBTC MOF, MgFe_2_O_4_ nanoparticles, and the fabricated CuBTC–MgFe_2_O_4_ MFCs, they were analysed using different characterisation techniques and depending on the outcome the pellets with 3 wt% magnetic content were selected for further oxygen studies. These pellets showed an adsorption capacity of 0.3 mmol g^−1^ at 1 bar pressure at 298 K. When they were exposed to a magnetic field of 31 mT at 269 kHz; the MgFe_2_O_4_ nanoparticles attained a temperature rise to 86 °C causing full desorption of the oxygen molecules. The composite exhibited good thermal stability and excellent cyclability by maintaining its adsorption properties over three continuous adsorption–desorption cycles. Oxygen storage and supply using ambient temperature processes can be made simpler, efficient, safer, and considerably less complex using cyclable MOFs. This, accompanied by the energy-efficient MISA process, can revolutionize the safe storage, handling, transport, and on-demand supply of oxygen.

## Conflicts of interest

There are no conflicts of interest to declare with the publication of this manuscript.

## Supplementary Material

RA-010-D0RA07068H-s001

## References

[cit1] Allam R. J. (2009). Energy Procedia.

[cit2] Dobson M. (2001). Int. J. Tuberc. Lung Dis..

[cit3] Emsley J. , Nature's building blocks: an AZ guide to the elements, Oxford University Press, 2011

[cit4] Gulcay E., Erucar I. (2019). Ind. Eng. Chem. Res..

[cit5] Dobson M. (1991). Anaesthesia.

[cit6] Arnold E., Bruton A., Donovan-Hall M., Fenwick A., Dibb B., Walker E. (2011). BMC Pulm. Med..

[cit7] Dubois A. , Bodelin P. and Vigor X., *US Pat.*, 6, 520, 176, 18 Feb 2003

[cit8] Moghadam P. Z., Islamoglu T., Goswami S., Exley J., Fantham M., Kaminski C. F., Snurr R. Q., Farha O. K., Fairen-Jimenez D. (2018). Nat. Commun..

[cit9] DeCoste J. B., Weston M. H., Fuller P. E., Tovar T. M., Peterson G. W., LeVan M. D., Farha O. K. (2014). Angew. Chem., Int. Ed..

[cit10] Weston M. H. , *US Pat.*, 20150105250, April 16, 2015

[cit11] Sadiq M. M., Suzuki K., Hill M. R. (2018). Chem. Commun..

[cit12] Falcaro P., Lapierre F., Marmiroli B., Styles M., Zhu Y., Takahashi M., Hill A. J., Doherty C. M. (2013). J. Mater. Chem. C.

[cit13] Mason J. A., Veenstra M., Long J. R. (2014). Chem. Sci..

[cit14] Bazer-Bachi D., Assié L., Lecocq V., Harbuzaru B., Falk V. (2014). Powder Technol..

[cit15] Kim J., Cho H.-Y., Ahn W.-S. (2012). Catal. Surv. Asia.

[cit16] Mendes R. F., Almeida Paz F. A. (2015). Inorg. Chem. Front..

[cit17] Peterson G. W., Britt D. K., Sun D. T., Mahle J. J., Browe M., Demasky T., Smith S., Jenkins A., Rossin J. A. (2015). Ind. Eng. Chem. Res..

[cit18] Bloch E. D., Queen W. L., Krishna R., Zadrozny J. M., Brown C. M., Long J. R. (2012). Science.

[cit19] Li H., Wang K., Sun Y., Lollar C. T., Li J., Zhou H.-C. (2018). Mater. Today.

[cit20] Li J.-R., Kuppler R. J., Zhou H.-C. (2009). Chem. Soc. Rev..

[cit21] Li J.-R., Sculley J., Zhou H.-C. (2011). Chem. Rev..

[cit22] Parkes M. V., Sava Gallis D. F., Greathouse J. A., Nenoff T. M. (2015). J. Phys. Chem. C.

[cit23] Queen W. L., Bloch E. D., Brown C. M., Hudson M. R., Mason J. A., Murray L. J., Ramirez-Cuesta A. J., Peterson V. K., Long J. R. (2012). Dalton Trans..

[cit24] Gangu K. K., Maddila S., Mukkamala S. B., Jonnalagadda S. B. (2016). Inorg. Chim. Acta.

[cit25] Ke F., Yuan Y.-P., Qiu L.-G., Shen Y.-H., Xie A.-J., Zhu J.-F., Tian X.-Y., Zhang L.-D. (2011). J. Mater. Chem..

[cit26] Yaghi O. M., Li G., Li H. (1995). Nature.

[cit27] Furukawa H., Cordova K. E., O'Keeffe M., Yaghi O. M. (2013). Science.

[cit28] Eddaoudi M., Moler D. B., Li H., Chen B., Reineke T. M., O'Keeffe M., Yaghi O. M. (2001). Acc. Chem. Res..

[cit29] Doherty C. M., Buso D., Hill A. J., Furukawa S., Kitagawa S., Falcaro P. (2014). Acc. Chem. Res..

[cit30] Hendon C. H., Rieth A. J., Korzyński M. D., Dincă M. (2017). ACS Cent. Sci.

[cit31] Li J.-R., Kuppler R. J., Zhou H.-C. (2009). Chem. Soc. Rev..

[cit32] Xiao D. J., Gonzalez M. I., Darago L. E., Vogiatzis K. D., Haldoupis E., Gagliardi L., Long J. R. (2016). J. Am. Chem. Soc..

[cit33] Murray L. J., Dinca M., Yano J., Chavan S., Bordiga S., Brown C. M., Long J. R. (2010). J. Am. Chem. Soc..

[cit34] Southon P. D., Price D. J., Nielsen P. K., McKenzie C. J., Kepert C. J. (2011). J. Am. Chem. Soc..

[cit35] Bloch E. D., Murray L. J., Queen W. L., Chavan S., Maximoff S. N., Bigi J. P., Krishna R., Peterson V. K., Grandjean F., Long G. J., Smit B., Bordiga S., Brown C. M., Long J. R. (2011). J. Am. Chem. Soc..

[cit36] Bloch E. D., Queen W. L., Hudson M. R., Mason J. A., Xiao D. J., Murray L. J., Flacau R., Brown C. M., Long J. R. (2016). Angew. Chem., Int. Ed..

[cit37] Xiao D. J., Gonzalez M. I., Darago L. E., Vogiatzis K. D., Haldoupis E., Gagliardi L., Long J. R. (2016). J. Am. Chem. Soc..

[cit38] Nagar H., Vadthya P., Prasad N. S., Sridhar S. (2015). RSC Adv..

[cit39] Melag L., Sadiq M. M., Smith S. J. D., Konstas K., Suzuki K., Hill M. R. (2019). J. Mater. Chem. A.

[cit40] Sugimoto H., Nagayama T., Maruyama S., Fujinami S., Yasuda Y., Suzuki M., Uehara A. (1998). Bull. Chem. Soc. Jpn..

[cit41] Moeljadi A. M. P., Schmid R., Hirao H. (2016). Can. J. Chem..

[cit42] Märcz M., Johnsen R. E., Dietzel P. D. C., Fjellvåg H. (2012). Microporous Mesoporous Mater..

[cit43] Thorseth M., Tornow C., Tse E., Gewirth A. (2013). Coord. Chem. Rev..

[cit44] Gonen S., Elbaz L. (2018). Curr. Opin. Electrochem..

[cit45] Gonen S., Lori O., Cohen-Taguri G., Elbaz L. (2018). Nanoscale.

[cit46] Lu X. F., Xia B. Y., Zang S.-Q., Lou X. W. (2020). Angew. Chem., Int. Ed..

[cit47] Jiang M., Li L., Zhu D., Zhang H., Zhao X. (2014). J. Mater. Chem. A.

[cit48] Skoulidas A. I. (2004). J. Am. Chem. Soc..

[cit49] Liu J., Wang Y., Benin A. I., Jakubczak P., Willis R. R., LeVan M. D. (2010). Langmuir.

[cit50] Al-Janabi N., Hill P., Torrente-Murciano L., Garforth A., Gorgojo P., Siperstein F., Fan X. (2015). Chem. Eng. J..

[cit51] Sun B., Kayal S., Chakraborty A. (2014). Energy.

[cit52] Gutiérrez-Sevillano J. J., Vicent-Luna J. M., Dubbeldam D., Calero S. (2013). J. Phys. Chem. C.

[cit53] Yang Q., Xue C., Zhong C., Chen J. F. (2007). AIChE J..

[cit54] Hulvey Z., Lawler K. V., Qiao Z., Zhou J., Fairen-Jimenez D., Snurr R. Q., Ushakov S. V., Navrotsky A., Brown C. M., Forster P. M. (2013). J. Phys. Chem. C.

[cit55] Rubio-Martinez M., Batten M. P., Polyzos A., Carey K.-C., Mardel J. I., Lim K.-S., Hill M. R. (2014). Sci. Rep..

[cit56] Liang Z., Marshall M., Chaffee A. L. (2009). Energy Fuels.

[cit57] Travlou N. A., Singh K., Rodríguez-Castellón E., Bandosz T. J. (2015). J. Mater. Chem. A.

[cit58] Hosseini M., Zeinali S., Sheikhi M. (2016). Sens. Actuators, B.

[cit59] Sadiq M. M., Li H., Hill A. J., Falcaro P., Hill M. R., Suzuki K. (2016). Chem. Mater..

[cit60] Li H., Sadiq M. M., Suzuki K., Ricco R., Doblin C., Hill A. J., Lim S., Falcaro P., Hill M. R. (2016). Adv. Mater..

[cit61] Huang B. L., McGaughey A. J. H., Kaviany M. (2007). Int. J. Heat Mass Transfer.

[cit62] Huang B. L., Ni Z., Millward A., McGaughey A. J. H., Uher C., Kaviany M., Yaghi O. (2007). Int. J. Heat Mass Transfer.

[cit63] Zhang X., Jiang J. (2013). J. Phys. Chem. C.

[cit64] Li H., Sadiq M. M., Suzuki K., Doblin C., Lim S., Falcaro P., Hill A. J., Hill M. R. (2016). J. Mater. Chem. A.

[cit65] Li H., Sadiq M. M., Suzuki K., Falcaro P., Hill A. J., Hill M. R. (2017). Chem. Mater..

[cit66] Sadiq M. M., Rubio-Martinez M., Zadehahmadi F., Suzuki K., Hill M. R. (2018). Ind. Eng. Chem. Res..

[cit67] Ricco R., Konstas K., Styles M. J., Richardson J. J., Babarao R., Suzuki K., Scopece P., Falcaro P. (2015). J. Mater. Chem. A.

[cit68] Ricco R., Malfatti L., Takahashi M., Hill A. J., Falcaro P. (2013). J. Mater. Chem. A.

[cit69] Kolhatkar A., Jamison A., Litvinov D., Willson R., Lee T. (2013). Int. J. Mol. Sci..

[cit70] Bañobre-López M., Teijeiro A., Rivas J. (2013). Rep. Pract. Oncol. Radiother..

[cit71] Harris I., Williams A. (2009). Mater. Sci. Eng., C.

[cit72] Rubio-Martinez M., Avci-Camur C., Thornton A. W., Imaz I., Maspoch D., Hill M. R. (2017). Chem. Soc. Rev..

[cit73] Maensiri S., Sangmanee M., Wiengmoon A. (2009). Nanoscale Res. Lett..

[cit74] Liu X.-M., Xie L.-H., Wu Y. (2020). Inorg. Chem. Front..

[cit75] Bakoglidis K., Simeonidis K., Sakellari D., Stefanou G., Angelakeris M. (2012). IEEE Trans. Magn.

[cit76] Sun S., Zeng H., Robinson D. B., Raoux S., Rice P. M., Wang S. X., Li G. (2004). J. Am. Chem. Soc..

[cit77] Deatsch A. E., Evans B. A. (2014). J. Magn. Magn. Mater..

[cit78] Ebrahimi M. (2016). J. Magn. Magn. Mater..

[cit79] Yang S. J., Choi J. Y., Chae H. K., Cho J. H., Nahm K. S., Park C. R. (2009). Chem. Mater..

[cit80] Gelfand B. S., Shimizu G. K. H. (2016). Dalton Trans..

[cit81] Bouson S., Krittayavathananon A., Phattharasupakun N., Siwayaprahm P., Sawangphruk M. (2017). R. Soc. Open Sci..

[cit82] Vishnyakov A., Ravikovitch P. I., Neimark A. V., Bülow M., Wang Q. M. (2003). Nano Lett..

[cit83] Lin K.-S., Adhikari A. K., Ku C.-N., Chiang C.-L., Kuo H. (2012). Int. J. Hydrogen Energy.

[cit84] Lestari W. W. , Adreane M., Purnawan C., Fansuri H., Widiastuti N. and Rahardjo S. B., 2016

[cit85] Mu B., Walton K. S. (2011). J. Phys. Chem. C.

[cit86] Bhardwaj S. K., Bhardwaj N., Kaur R., Mehta J., Sharma A. L., Kim K.-H., Deep A. (2018). J. Mater. Chem. A.

[cit87] Rudolf H., Silvio D., Matthias Z. (2010). Nanotechnology.

[cit88] Sung H. W. and Rudowicz C., arxiv preprint cond-mat/0210657, 2002

[cit89] Mason J. A., Sumida K., Herm Z. R., Krishna R., Long J. R. (2011). Energy Environ. Sci..

[cit90] Li H., Sadiq M. M., Suzuki K., Doblin C., Lim S., Falcaro P., Hill A. J., Hill M. R. (2016). J. Mater. Chem. A.

[cit91] Sadiq M. M., Rubio-Martinez M., Zadehahmadi F., Suzuki K., Hill M. R. (2018). Ind. Eng. Chem. Res..

[cit92] Twort A. C. , Water supply, London: Arnold/IWA Pub., London, 5th edn, 2000

