# Supplementary material for: Performance evaluation of CuBTC composites for room temperature oxygen storage
Source: RSC Adv. 2020 Nov 10;10(67):40960–8. doi: 10.1039/d0ra07068h (PMC9057710; doi:10.1039/d0ra07068h)
Supplement: RA-010-D0RA07068H-s001 [file RA-010-D0RA07068H-s001.pdf]

Electronic Supplementary Material (ESI) for RSC Advances.
This journal is © The Royal Society of Chemistry 2020

Supporting Information

Performance evaluation of CuBTC composites for room temperature Oxygen storage.

Leena Melag, [†] M. Munir Sadiq, [‡] Kristina Konstas [‡], Farnaz Zadehahmadi, Kiyonori Suzuki, [*‖] and Matthew R. Hill, [*†‡]

[†]Department of Chemical Engineering, Monash University, Clayton, VIC 3168, Australia

[‖] Department of Materials Science and Engineering, Monash University, Clayton, VIC 3168, Australia

[‡] CSIRO, Private Bag 33, Clayton South MDC, VIC 3169, Australia

Powder X-Ray Diffraction and FTIR spectroscopy of CuBTC MOF:

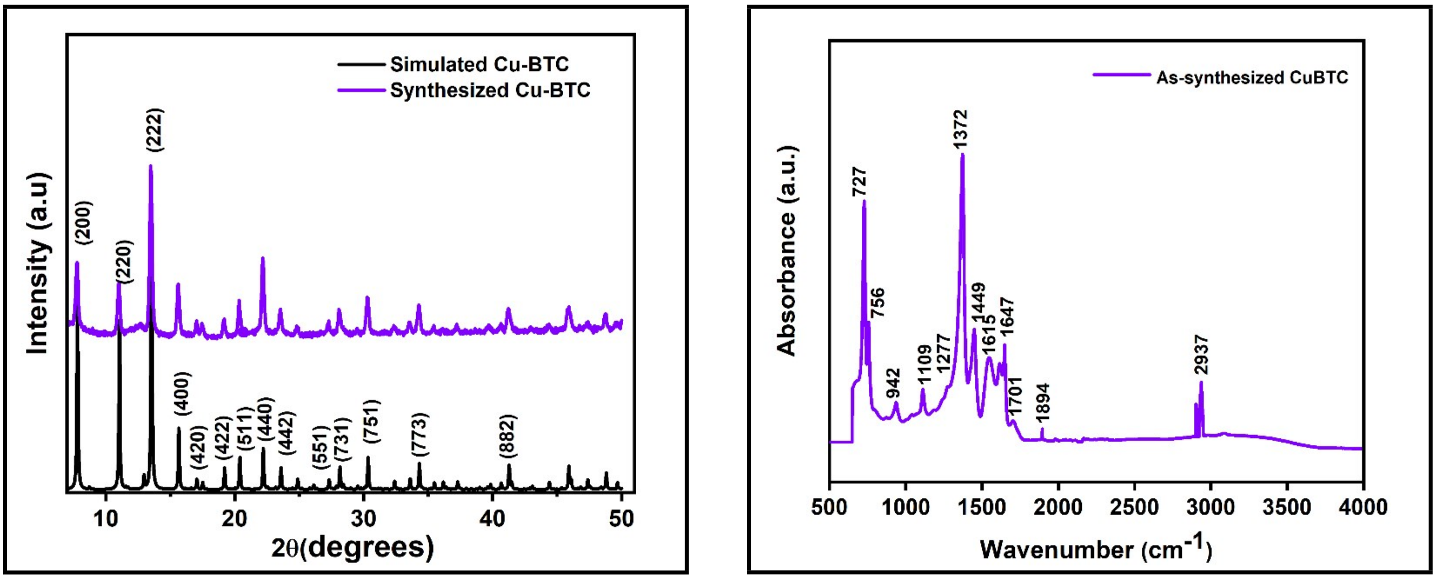

a) b)

Figure. S1-a) The XRD patterns of the synthesised CuBTC MOF are a perfect match with the simulated XRD pattern of CUBTC, b) The FTIR spectra of the synthesised CuBTC to detect the functional groups in the sample.

The XRD pattern of the synthesised Cu-BTC is shown in Fig S1, and it is characteristic of a face-centred cubic (FCC) CuBTC crystal lattice and the reflection peaks of (200), (220), (222), (400), (420), (511),(440),(442),(551),(731),(751),(773), and (882) match the reflections of the simulated CuBTC pattern. Fourier-Transform Infrared (FTIR) spectra for all samples were collected using a Thermo Scientific NICOLET 6700 FT-IR and was used to detect the functional groups in the samples. The FTIR spectrum of samples match closely with the ones reported in the literature [1, 2] with the significant peaks observed at 580-727 cm−1 attributed to the vibrational bond involving the Cu-O, the peaks around 1449 and 1372 cm−1 due to the symmetric stretching modes and the one around 1647 cm−1 due to the asymmetric stretching of carboxylate groups in the BTC linker.

Powder X-Ray Diffraction and SEM on $MgFe_2O_4$ nanoparticles:

As shown in Fig. S2, a), the XRD pattern of the synthesised $MgFe_2O_4$ nanoparticles with diffraction peaks of planes (2 2 0), (3 1 1), (4 0 0), (5 1 1) and (4 4 0), for a cubic spinel $MgFe_2O_4$ phase, matches the standard powder diffraction data (ICSD #00-036-0398) of the $MgFe_2O_4$ phase from literature[3]. The mean crystallite size of 10.2 nm was calculated from the peak broadening of the diffraction planes and used in the Scherrer's equation, $d = K\lambda/(\beta \cos\theta)$, where d is the crystallite size, λ is the wavelength of the X-ray radiation (Co-1.78897 Å), K is a shape factor with values in between 0.9-1, θ is the Braggs peak in degrees and β is the line broadening (full width at half maximum). Scanning electron microscope (SEM) analysis on the samples was carried out on a JEOL JSM-7001F microscope and, as shown in Fig. S2, b), the images show uniform, spherical, and agglomerated $MgFe_2O_4$ nanospheres with a diameter of about 150-160 nm.

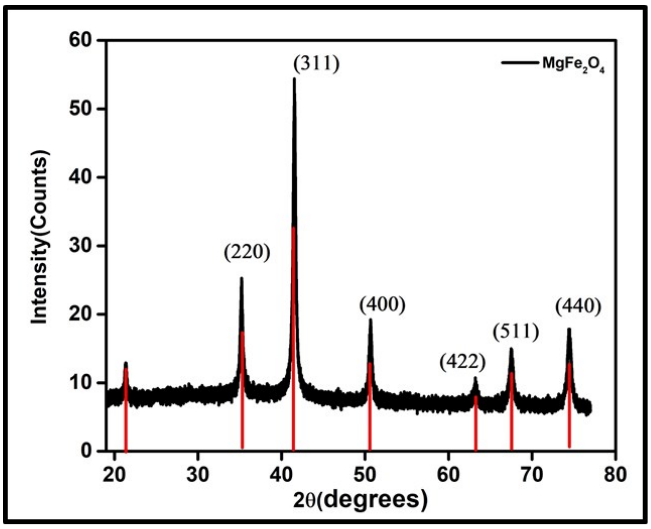

(a)

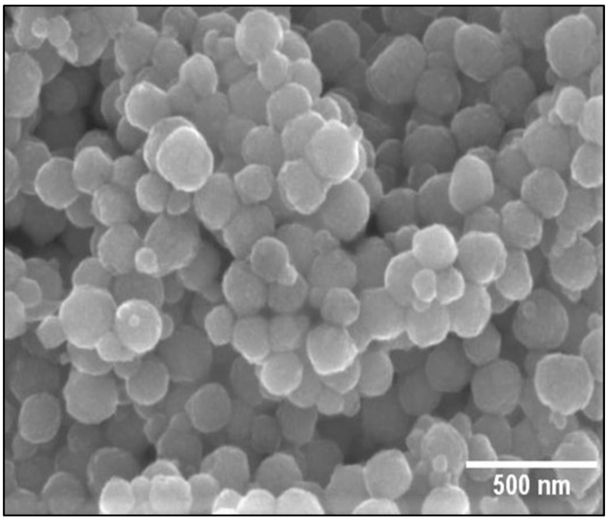

(b)

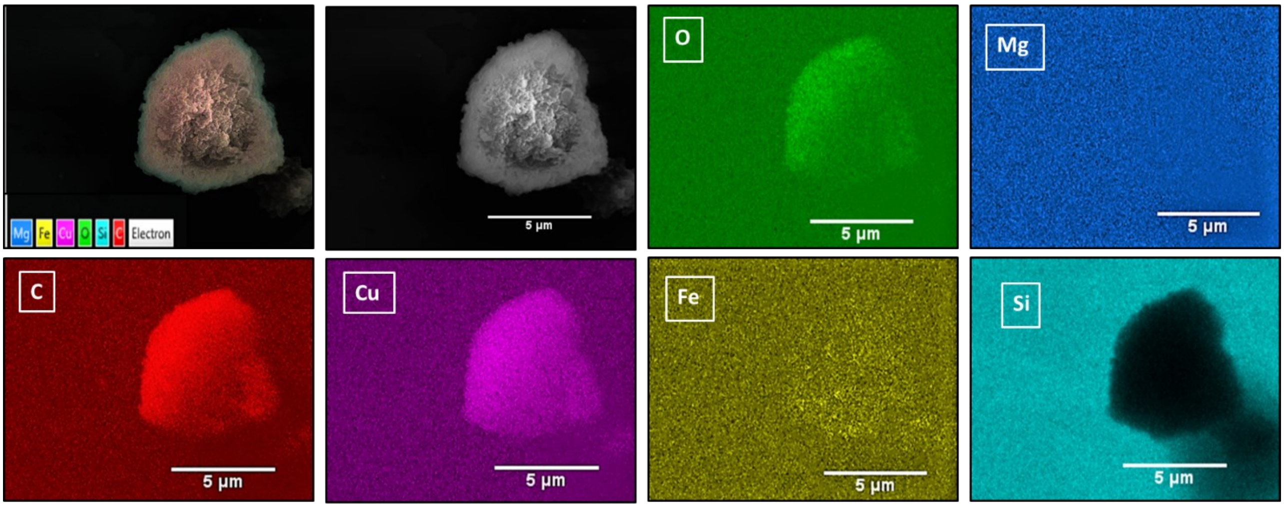

c)

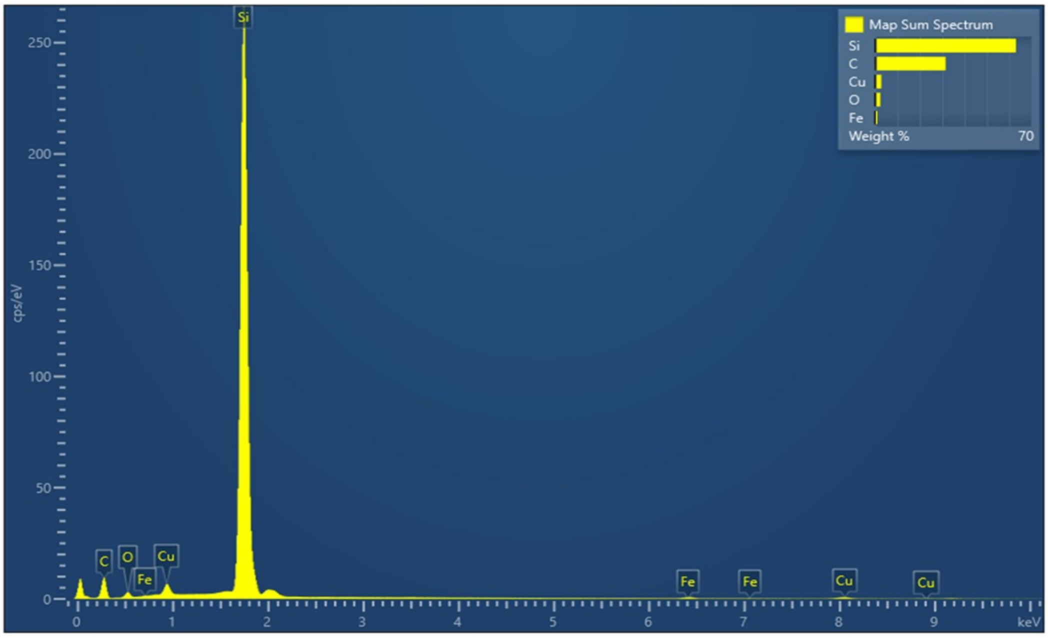

d)

Element	Wt.%
C	31.51
O	2.22
Si	63.08
Fe	0.5
Cu	2.69
Total:	100

e)

Figure. S2: a) XRD patterns for a cubic spinel $MgFe_2O_4$ phase that matches the standard powder diffraction data (ICSD #00-036-0398), b) SEM images showing the spherical, uniform $MgFe_2O_4$ nanospheres, c) SEM-EDS mapping of the 3 wt.% CuBTC-$MgFe_2O_4$ composite showing the elemental distribution, d) & e) EDX spectrum of the composite displaying its elemental composition.

Vibrating Sample Magnetometer:

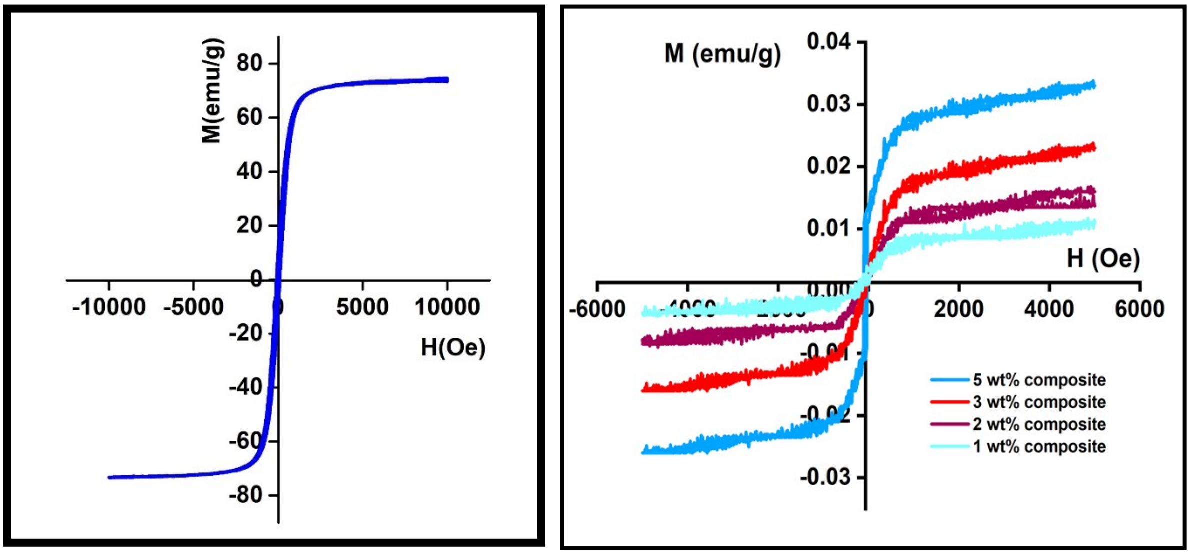

a) b)

Figure. S3 -a) The magnetic hysteresis loops of bare $MgFe_2O_4$ showing a saturation magnetisation of 65 emu/g and b) the magnetic hysteresis loops of the CuBTC-$MgFe_2O_4$ composites with varying magnetic contents.

The saturation magnetisation (Ms), coercivity (Hc), and magnetisation curves were studied on a vibrating sample magnetometer (VSM, RIKEN DENSHI) operated at room temperature. The samples are prepared by embedding the magnetic nanoparticles in an epoxy resin and hardener mix, and once it was all set, the measurements were carried out. The results of the $MgFe_2O_4$ nanospheres synthesised show a saturation magnetisation of 65 emu/g, and with MFCs, the saturation magnetisation values were higher for composites with higher concentrations of magnetic nanoparticles with 3 wt.% CuBTC-$MgFe_2O_4$ composite showing 0.025 emu/g.

Thermo-magneto gravimetric analysis

Curie point is a temperature at which magnetic materials undergo loss of their magnetic properties and become paramagnetic. This sharp change in magnetic properties is measured by measuring the samples' weight changes as a function of temperature. The curie temperature of the $MgFe_2O_4$ nanoparticles was determined using the Thermo-magneto gravimetric analysis, which is a very efficient technique to ascertain the magnetic transitions in the nanoparticles. A weighed amount of $MgFe_2O_4$ nanoparticles were heated from room temperature to 800 °C in a nitrogen atmosphere using a heating rate of 40 °C /min, and the Curie temperature was found to be Tc=566 °C.

Specific Absorption Rate (SAR):

The rate at which the magnetic nanoparticles will absorb the magnetic energy and convert it into thermal energy can be predicted using a Specific adsorption rate (SAR). Apart from the diameter, shape, and composition of the nanoparticles, SAR is strongly governed by the frequency of the applied magnetic field. High SAR values are preferred because it means higher heating efficiency. Magnetic nanoparticles with higher SAR values are ideal for an efficient MISA process.

SAR is calculated by dispersing the particles in a liquid medium and the measuring:

$$\text{SAR} = C_s \frac{m_s}{m_m} \left(\frac{dT}{dt}\right)_{t=0}$$

Where Cs is the specific heat capacity of suspension, dT/dt at t =0 is the initial gradient of the heating curve, ms, and mm are the specific masses of the suspension and magnetic particles, respectively. The units of SAR are watts per kilogram (W/kg). The SAR of the synthesised $MgFe_2O_4$ nanoparticles was calculated using the temperature rise profile by dispersing 10 mg of the $MgFe_2O_4$ nanoparticles in 1ml of water and exposing it to a 25 mT magnetic field. As seen from figure S4-b, the temperature stabilised at 74 ^{0}C, and the SAR was calculated to be 130.6 W/g.

Temperature Rise Profile:

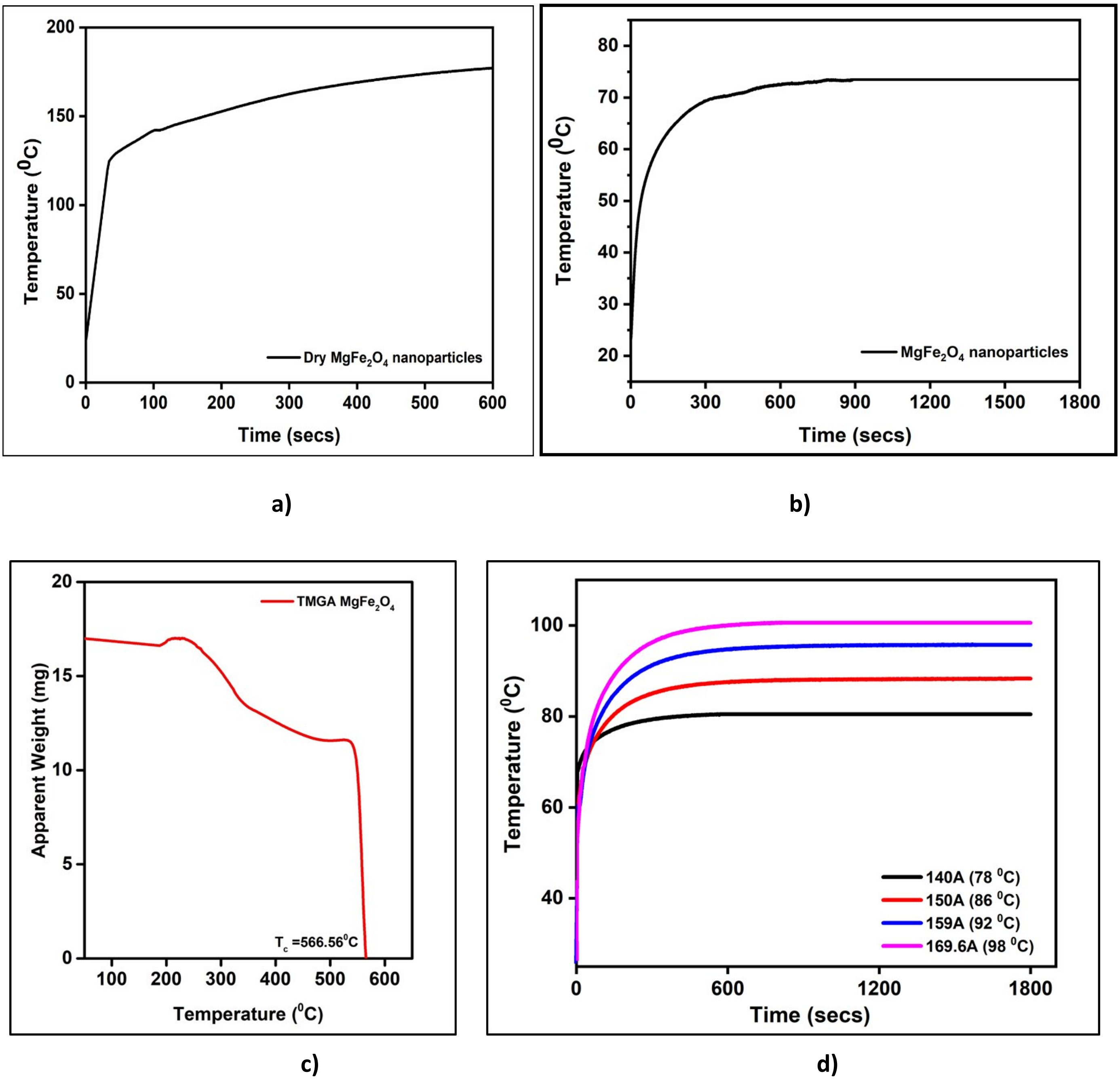

Figure. S4 -: a) Temperature rise profile of dry $MgFe_2O_4$ nanoparticles, b): Temperature rise profile of 10 mg of the synthesised $MgFe_2O_4$ nanoparticles dispersed in 1 ml of water at 25 mT, c) The curie point of the MgFe2O4 was measured to be 566 $^\circ$C using the thermo-magneto gravimetric analysis, d) Temperature rise attained by the 3 wt. % CuBTC-MgFe2O4 composites when exposed to varying magnetic fields.

Porosity and Surface Area Measurements:

Micrometric ASAP 2420 machine was used to measure the porosity and surface area properties of the sample. Initially, empty BET sample tubes were weighed, then they were filled with the CuBTC samples sealed with Transeals caps and then weighed again. The samples were heated under vacuum to 140 ^{0}C for 24 hrs and reweighed again to calculate the actual mass of the degassed samples. The sample tubes were then attached to the ASAP ports for N_2 studies at 77K temperatures. The effect of the addition of binders and effect of binders as well as the magnetic nanoparticles is studied using the N_2 uptake isotherms.

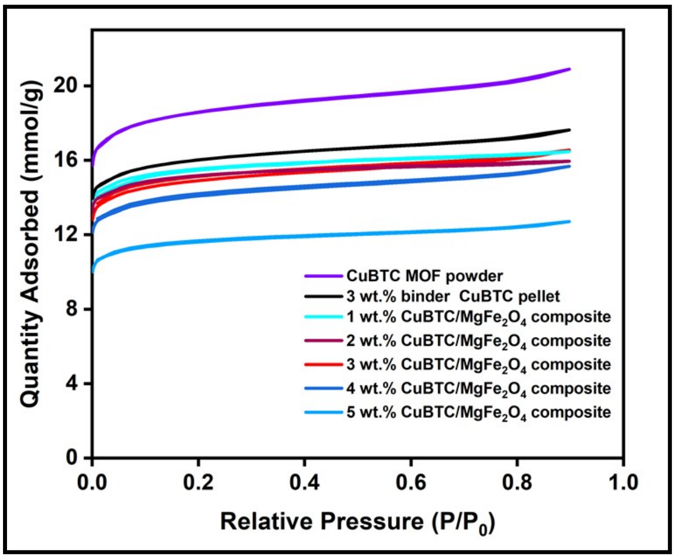

a)

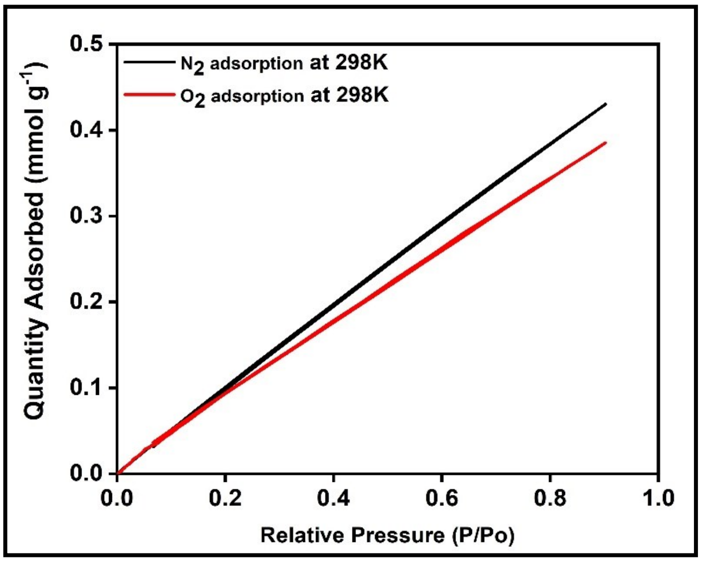

b)

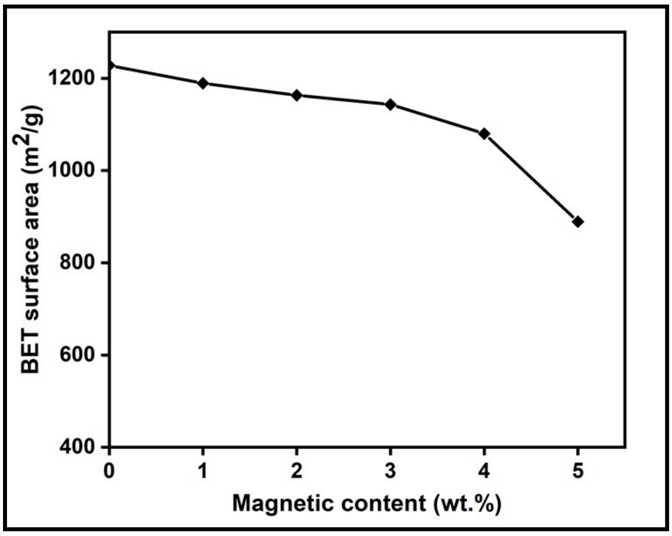

c)

Figure S5: The BET surface area measurements of the composites by Nitrogen adsorption at 77 K, b) The Nitrogen and oxygen uptake by the 3 wt.% CuBTC-$MgFe_2O_4$ composite at 298 K, c) The effect of increasing concentrations of magnetic content on the BET area of the composites.

The concentration of nanoparticles in the composite significantly influences its adsorption and desorption properties. Composites with higher magnetic content can lower its adsorption capacities but provide higher heating rates to achieve desorption whereas composites with

lower magnetic content can demonstrate high adsorption capacities but insufficient heating abilities. Using 3 wt.% binder for pelletising the composites, the effect of varying magnetic contents on the surface area properties of the MFC were studied.

**MOF / Composite**	**BET Surface area (m^2/g)**
CuBTC MOF powder	1495
(CuBTC MOF + 3 wt.% binder)	1228
(CuBTC MOF + 3 wt.% binder) + 1 wt.% $MgFe_2O_4$ NPs	1189
(CuBTC MOF + 3 wt.% binder) + 2 wt.% $MgFe_2O_4$ NPs	1164
(CuBTC MOF + 3 wt.% binder) + 3 wt.% $MgFe_2O_4$ NPs	1143
(CuBTC MOF + 3 wt.% binder) + 4 wt.% $MgFe_2O_4$ NPs	1080
(CuBTC MOF + 3 wt.% binder) + 5 wt.% $MgFe_2O_4$ NPs	889

Table S1: The BET surface areas of the CuBTC MOF, the CuBTC pellet using 3 wt.% binder and the effect of the addition of 2 wt.%, 3 wt.%, 4 wt.% and 5 wt.% $MgFe_2O_4$ nanoparticles on the surface area properties of the CuBTC/ $MgFe_2O_4$ composites.

As observed from Table S1 and Figure 2., the initial BET surface area of 1495 m^2/g the CuBTC MOF powder was reduced to 1228 m^2/g during the pelletising process using 3 wt.% binder. The surface area of the composites decreased further with additions of 1 wt.%, 2 wt.%, 3 wt.%, 4 wt.% and 5 wt.% $MgFe_2O_4$ nanoparticles to this mix.

Moisture stability of CuBTC MOF and composite:

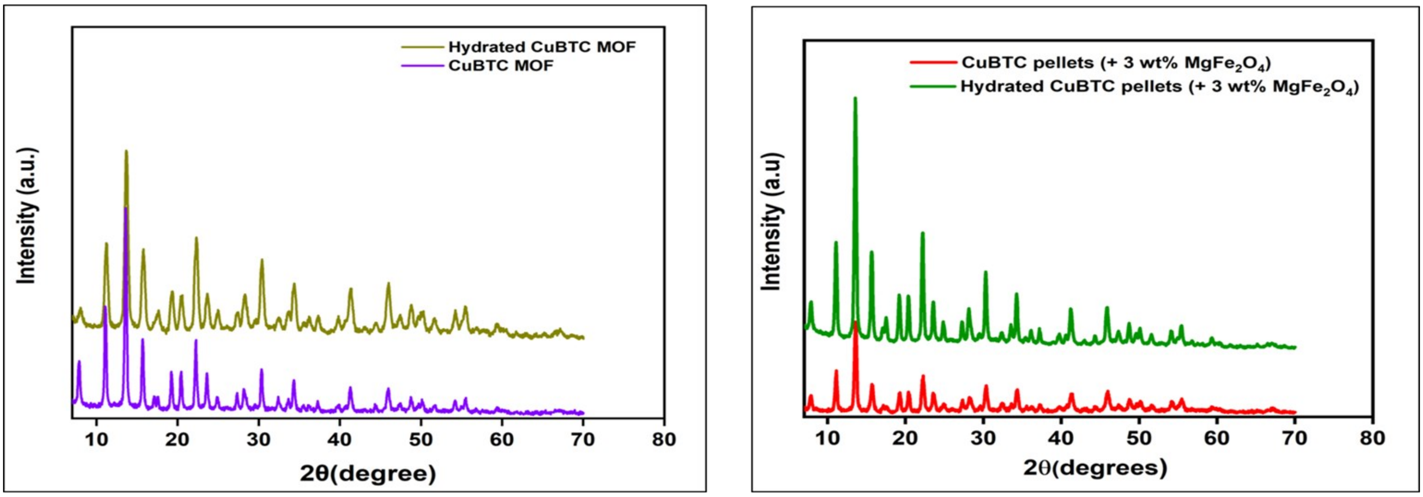

a) b)

Figure S6: XRD patterns for a) CuBTC MOF and b) the 3 wt.% CuBTC-$MgFe_2O_4$ composite before and after water vapour adsorption at 298 K.

Thermal Stability and Atmospheric Stability of the composites:

Thermal stabilities of the samples were studied by thermal gravimetric analysis (TGA) using weighed samples that were heated from 25 ^oC to 800 ^oC using a heating rate of 10 ^oC min^{-1}. As observed in Figure S5-a), the TGA of bare as-synthesised CuBTC MOF,

TGA of 3 wt.% CuBTC-$MgFe_2O_4$ composite pellets and TGA of the same sample after exposure of the environment for 30 days has been plotted. In all three samples, the two significant weight losses are observed, the first one corresponds to the evaporation of the solvent, and the second weight loss corresponds to the decomposition of MOF and or composites. Apart from the weight losses corresponding to the decomposition of the MOFs, the thermal stabilities of the CuBTC MOF and the 3 wt.% CuBTC-$MgFe_2O_4$ composite are very similar, indicating that the use of binders and the shaping procedure has not adversely affected its stability. As confirmed by other characterisation techniques, atmospheric exposure has reduced the thermal stability of the composite. The XRD of the exposed sample shows some peaks broadening corresponding to either the introduction of defects in the MOF framework or the slow degradation of the framework.

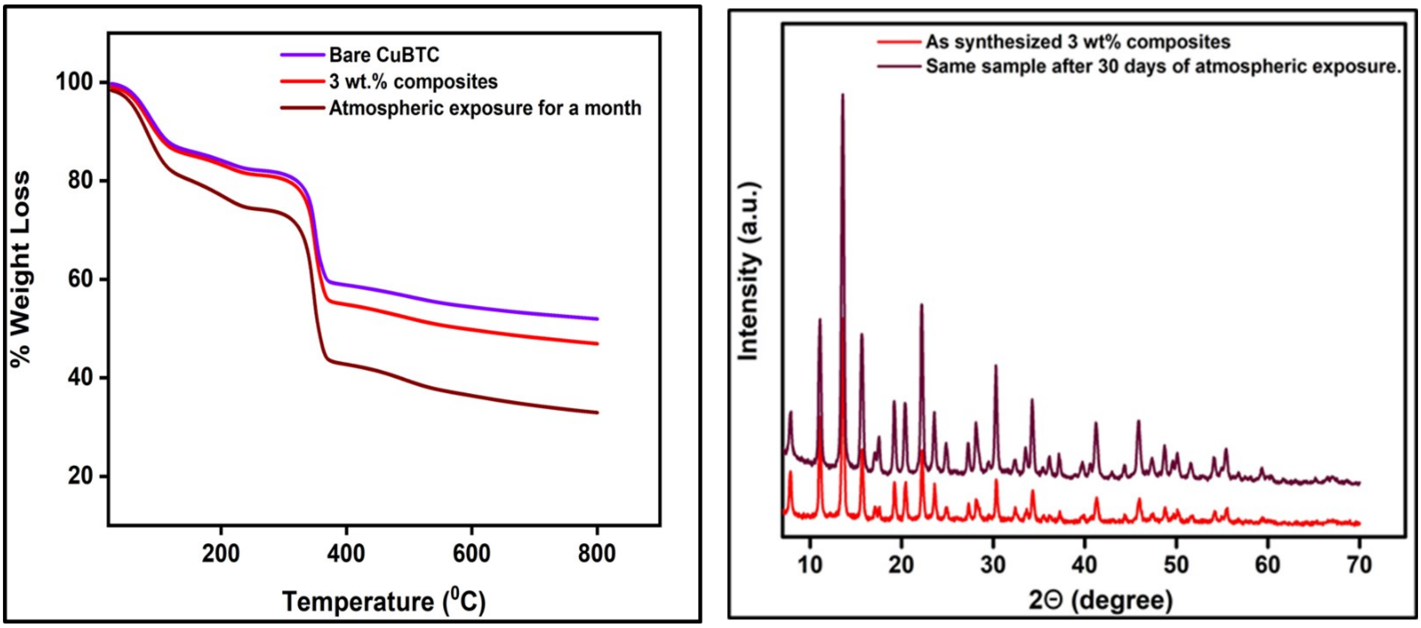

a) b)

Figure S7: a) TGA analysis of the synthesised CuBTC MOF, the 3 wt.% CuBTC-$MgFe_2O_4$ composite, and the 3 wt.% CuBTC-$MgFe_2O_4$ composite after exposure to atmosphere for 30 days, b) XRD of the 3 wt.% CuBTC-$MgFe_2O_4$ composite before and after atmospheric exposure.

Isosteric Heat of Adsorption:

The isosteric heats of adsorption, Qst, reveals the extent of interaction between the adsorbed molecules and the adsorbate under constant loading conditions. In the case of CuBTC MOF, this interaction that is dependant on the reactions at the exposed cationic Cu2+ sites and adsorption at the windows sites of the octahedral CuBTC cage is calculated to be -15.3 kJ mol-1 (Figure S6-b). This near-constant Qst curve was plotted using the adsorption data measured at 204 K, 273, and 298 K (Figure S6-a) and shows that irrespective of the low or loading conditions the binding energies remain the same.

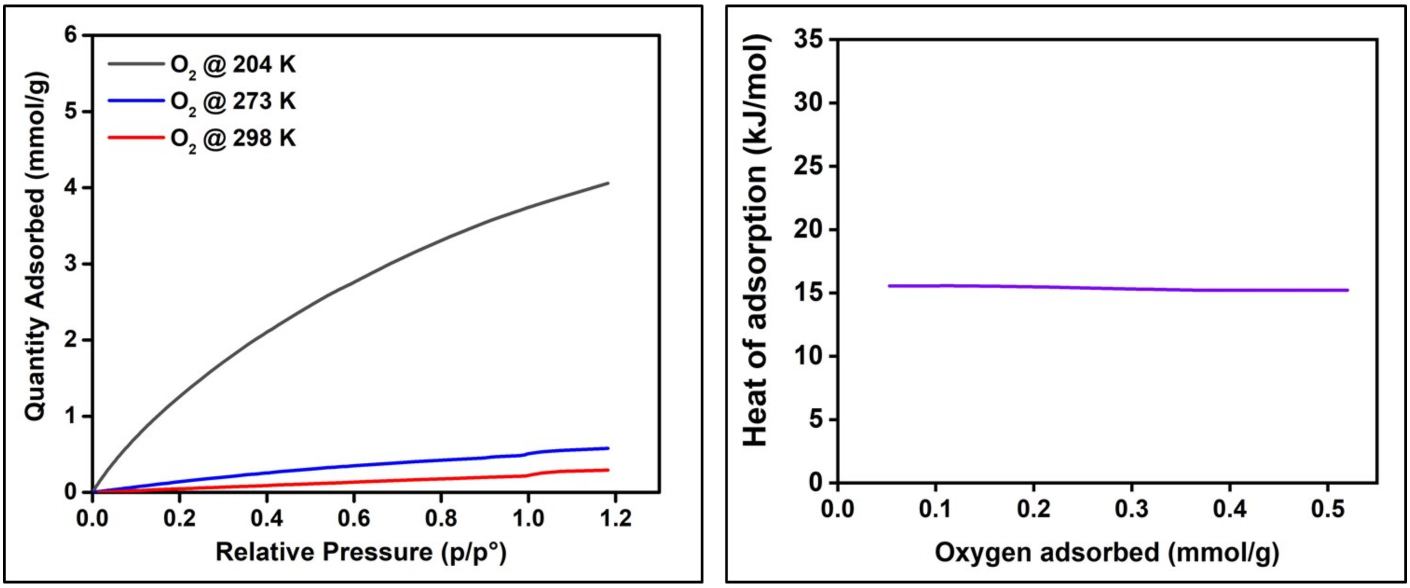

a) b)

Figure S8: a) Oxygen adsorption isotherm of CuBTC MOF at 204 K, 273 K, and 298 K, b) The heat of adsorption curve, Qst curve, plotted using the adsorption data measured at 204 K, 273 and 298 K.

Oxygen Adsorption Measurements & Triggered Release Experiments:

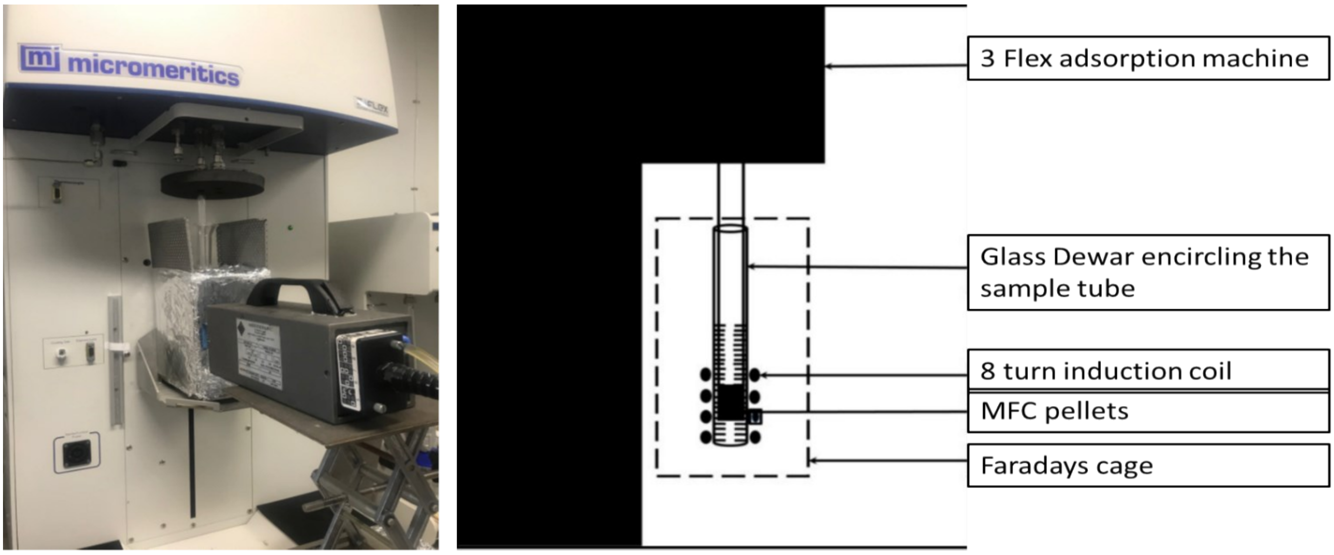

Figure S9-a) The set-up for oxygen adsorption measurements consists of the 3Flex machine and the EASY HEAT Ambrell induction machine with an 8 turn coil that is strategically placed around the sample tube so that the sample is uniformly exposed to the magnetic field, b) the graphical representation of the triggered release experiments.

The oxygen adsorption studies were carried out on a Micro metrics 3Flex surface and catalyst characterisation instrument. The triggered release set-up consisted of the activated and weighed CuBTC pellets filled in a 3flex tube and attached to the 3flex machine while a slightly wider glass dewar encircled it.

Regeneration Energy using the MISA process:

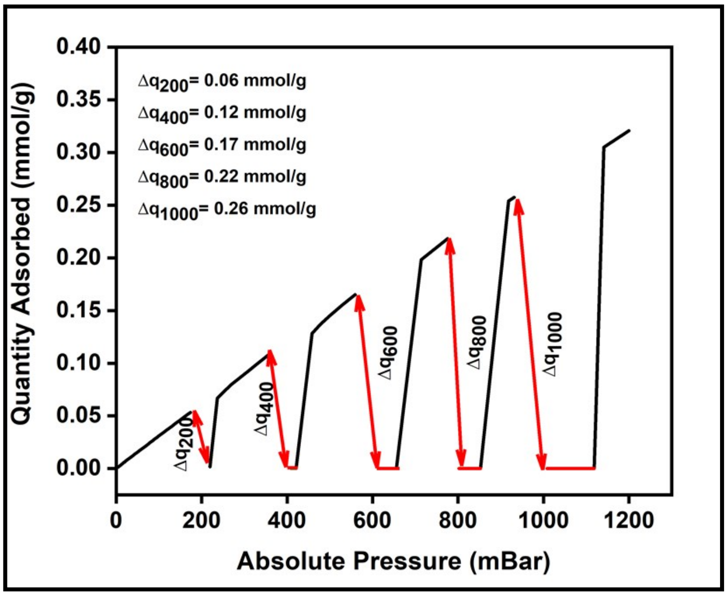

Figure S10: Working capacity is the difference in oxygen adsorbed under normal adsorption conditions, and the oxygen retained after desorption.

At this lab scale set-up, it is not possible to directly determine energy use for the process, but the energy calculations for the estimated regeneration energy (Qthermal) of our composite per kilogram of O_2 captured can be calculated. Specific regeneration energy (Qthermal)

is reported as the sum of the energy required to heat the adsorbent to the desorption temperature, and the energy required to desorb bound gas species from the adsorbent, according to the equation:

$$Q_{thermal} = \frac{c_p\, m_{sorbent}\Delta T + (\Delta h O_2 \Delta q O_2)}{m O_2}$$

Where,

Cp = specific heat capacity of adsorbent ($jg^{-1}K^{-1}$)

msorbent = mass of adsorbent (g)

ΔT = Temperature difference between adsorption and desorption conditions (K)

Δh = heat of adsorption (kJmol-1)

Δq = working capacity, it can be defined as the difference between the O_2 loadings at adsorption and O_2 loadings at the end of desorption.

$mO_2 =$ mass of oxygen adsorbed at that pressure (g)

Applying this equation to the values in Table S1, the energy required to regenerate the amount of oxygen captured at 200 mbar, 400 bar, 800 mbar and 1000 mbar is calculated, and the values are:

**Magnetic field Strength**	**25 (mT)**					**31(mT)**					**33(mT)**				
**Regeneration Pressure (mBar)**	**200**	**400**	**600**	**800**	**1000**	**200**	**400**	**600**	**800**	**1000**	**200**	**400**	**600**	**800**	**1000**
**Adsorption Temperature (°C)**	25	25	25	25	25	25	25	25	25	25	25	25	25	25	25
**Regeneration Temperature (°C)**	86	86	86	86	86	92	92	92	92	92	96	96	96	96	96
**Regeneration Time (s)**	362	360	359	360	359	300	300	300	300	300	180	180	180	180	180
**Amount of O_2 desorbed (mmol/g)**	0.04	0.10	0.14	0.19	0.23	0.06	0.12	0.17	0.22	0.26	0.06	0.12	0.17	0.22	0.26
**Regeneration Energy (Q) (MJ/kg)**	22.71	11.26	7.74	6.05	5.14	24.90	12.34	8.4	6.6	5.6	27.07	13.43	9.23	7.22	6.13
**Energy Consumption (kWh/kg_{O2})**	1.47	0.71	0.50	0.37	0.31	0.65	0.37	0.24	0.18	0.15	0.51	0.27	0.18	0.14	0.11

Table S2: The estimated regenerative energy and power calculations for 0.6 gms of 3 wt.% CuBTC-$MgFe_2O4$ composite pellets at 25mT, 31 mT, and 33mT.

To establish the energy efficiency of the MISA process, power calculations were done using a power meter to measure the power required by the induction coil at varying magnetic fields of 25 mT, 31 mT, and 33 mT. To determine the power required to regenerate a weighed amount of sample, these calculations were done without exposing the samples to the magnetic fields and with samples being exposed to the magnetic fields (Figure S11-a). The power required was calculated from the output readings from the power meter, the time required for the samples to desorb all the adsorbed molecules, and the mass of the desorbed molecules.

**Absolute pressure (mbar)**	**Regeneration energy (MJ/kg)** Using 31 mT magnetic field	
	**0.33 gms**	**0.66 gms**
200	22	24.9
400	11.2	12.34
600	7.7	8.4
800	5.8	6.6
1000	5.1	5.6

Table- S3: Estimated energy required to regenerate 0.33gms and 0.6 gms of 3 wt.% CuBTC-MgFe$_2$O4 composite pellets using magnetic fields of 31 mT.

Based on these calculations it was determined that using a magnetic field strength of 31 mT, that triggered a temperature rise of 86 ^{o}C in the 0.6gms of 3 wt.% CuBTC-MgFe$_2$O$_4$ composite, 0.65 kWh/kg$_{O2}$ power was consumed to desorb 0.3 mmol/g of adsorbed oxygen molecules. To understand how the weight of the sample influences the regeneration energies, using a magnetic field strength of 31 mT, the calculations were done using two samples weighing 0.33 gms and 0.64 gms each, and for 0.33 gms sample 5.1 MJ kg $^{-1}$ is required at 1000 mbar, and for 0.64 gms of the same sample, 5.6 MJ kg $^{-1}$ of energy is required at 1000 mbar(Figure S11-b).

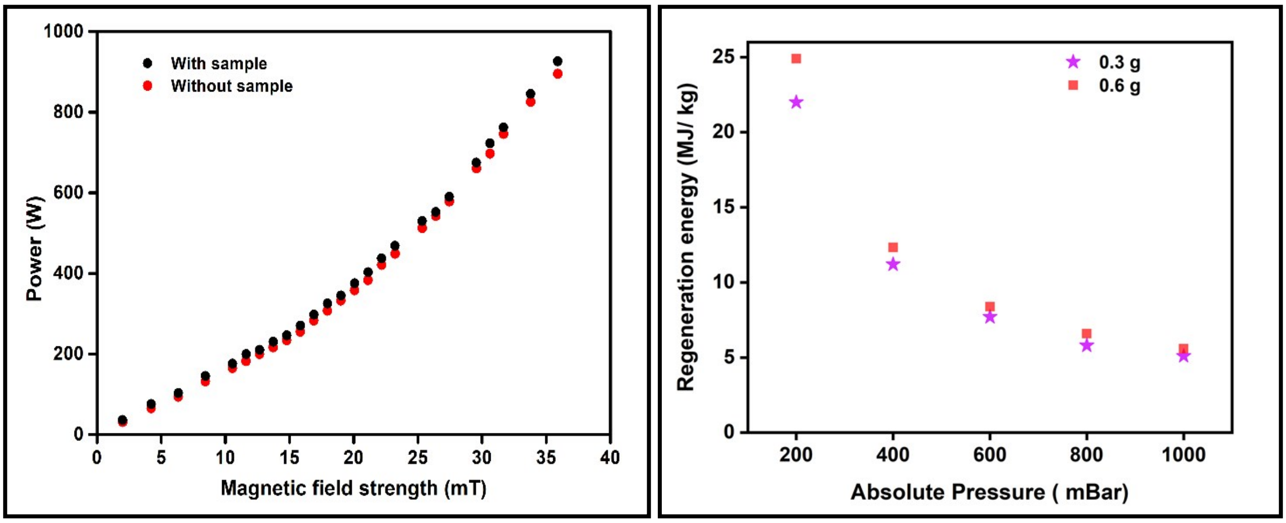

Figure S-11: a) Power calculations were done using the Magnetic induction set-up for power consumed with and without the sample. b) To study the regeneration energy required for different amounts of samples, 0.3 gms of the sample, and 0.6 gms of samples.

References:

1. S. Bouson, A. Krittayavathananon, N. Phattharasupakun, P. Siwayaprahm and M. Sawangphruk, *R. Soc. Open Sci*, 2017, **4**, 170654.

2. S. Homayoonnia and S. Zeinali, *Sens. Actuators, B*, 2016, **237**, 776-786.
3. S. Maensiri, M. Sangmanee, and A. Wiengmoon, *Nanoscale Res. Lett*, 2009, **4**, 221.
